# Integrating reaction norm models and genome-wide association analyses to reveal the genetic architecture and environmental sensitivity of sexual precocity in Nellore cattle

**DOI:** 10.1186/s12864-026-12547-8

**Published:** 2026-01-21

**Authors:** Eduarda da Silva Oliveira, Hinayah Rojas de Oliveira, Lucio Flavio Macedo Mota, Henrique Alberto Mulim, Milena Aparecida Ferreira Campos, João Barbosa da Silva Neto, Fernando Baldi

**Affiliations:** 1 São Paulo State University, School of Agriculture and Veterinary Science, Jaboticabal, SP 14884-900 Brazil; 2 Department of Animal Sciences, Purdue University, West Lafayette, Indiana 47907 USA; 3 School of Veterinary Medicine and Animal Science, Federal University of Bahia, Salvador, Bahia 40170-110 Brazil; 4 Department of Veterinary, Faculty of Animal Science and Food Engineering, University of São Paulo, Pirassununga, São Paulo Brazil

**Keywords:** Bos taurus indicus, Genotype-by-environment interaction, GWAS, Reproductive traits, Tropical environments

## Abstract

**Background:**

Reproductive efficiency is an important component of profitability and sustainability in beef cattle production, particularly in tropical environments where animals are routinely exposed to environmental stressors. This study aimed to identify environmentally sensitive SNPs associated with sexual precocity traits in Nellore cattle and characterize candidate genes and biological pathways regulating sexual precocity under variable environmental conditions. For this purpose, three sexual precocity indicators were analyzed; one in heifers (heifer early calving probability at 30 months, HC30), and two in young males (scrotal circumference at 365 days, SC365; age at puberty, APM). Reaction norm models were integrated with genome-wide association studies (GWASs) to identify genomic regions associated with both genetic potential (intercept) and environmental sensitivity (slope).

**Results:**

A total of 46 significant SNPs were identified across the three traits, with SC365 showing the highest genetic complexity (36 SNPs), followed by HC30 (7 SNPs), and APM (3 SNPs). The analyses revealed distinct genetic architectures among the traits. For HC30, candidate genes showed clear functional partitioning between those associated with baseline fertility (e.g., *ERBB4*,* SNAI2*) and those associated with environmental sensitivity (e.g., *TRIB1*,* NSMCE2*), with no overlap between intercept and slope components. In contrast, SC365 exhibited substantial genetic overlap, with five genes (i.e., *GRB14*,* SLC9A8*,* SPATA2*,* MGRN1*,* SEPTIN12*) significantly associated with both intercept and slope, indicating a robust trait with shared genetic control of baseline potential and environmental responsiveness. For APM, genetic associations were predominantly related to baseline potential, with genes involved in DNA repair (*CHEK2*), endoplasmic reticulum stress response (*XBP1*), and immune regulation (*TNIP3*). Functional enrichment analyses revealed trait-specific biological pathways, whereas QTL enrichment demonstrated biologically coherent overlaps with reproductive and metabolic traits.

**Conclusion:**

These findings demonstrate that sexual precocity traits exhibit distinct genetic architectures reflecting their underlying biological mechanisms and environmental responsiveness, providing valuable insights for developing climate-resilient breeding strategies in tropical beef cattle production systems.

**Supplementary Information:**

The online version contains supplementary material available at 10.1186/s12864-026-12547-8.

## Background

Global livestock production is increasingly challenged by more frequent and intense climate extremes, especially heatwaves, which impair animal performance and disproportionately affect tropical and subtropical systems [1]. In these regions, which are expected to account for a significant portion of future beef production, maintaining animal resilience is essential for the economic sustainability of the production system. In tropical production systems, Nellore cattle (*Bos taurus indicus*), the main beef breed in Brazil, are particularly relevant due to their inherited thermotolerance and greater parasite resistance compared to *Bos taurus taurus* under harsh environmental conditions [2]. Consequently, Nellore cattle have been extensively studied to assess the environmental effects on economically important traits, aiming to design more robust breeding programs [3–5].

Improving reproductive efficiency in males and females is crucial for enhancing the profitability and sustainability of production systems, particularly in tropical regions where animals are routinely exposed to environmental stressors [6, 7]. Selecting for sexual precocity reduces the age at first calving, extends lifetime productivity, increases the number of calves weaned and shortens the generation interval, resulting in substantial economic and environmental benefits [8, 9]. In Nellore cattle, key indicators of sexual precocity include heifer early calving probability at 30 months (HC30), scrotal circumference at 365 days (SC365), and age at puberty in males (APM). In Nellore cattle, heritability estimates for HC30 have ranged from 0.16 to 0.37 [10–12], from 0.37 to 0.42 for SC365 [13–15], and 0.30 for APM [16]. These traits are heritable and useful as selection criteria for early reproductive performance. Although SC365 and APM present heritability estimates comparable to those of many production traits, the realized selection response depends on the relative weight assigned to these traits in the breeding programs’ selection indices.

Despite significant advancements in selection methods over the years [17], further accelerating genetic gain for sexual precocity continues to be challenging because the trait combines a complex genetic architecture [12, 18–20] with strong environmental modulation [21]. A critical factor affecting the performance of Nellore cattle is the genotype-by-environment interaction (GxE), whereby genetic ranking shifts with environmental context due to environment-responsive gene expression [21–24]. Regionalized evaluations show that heritability for reproductive traits varies across Brazil, so sires identified as superior in one region may not perform similarly elsewhere [25]. Re-ranking has also been reported for growth across production systems (feedlot vs. pasture) [26, 27] and for feed-efficiency traits [28]. Collectively, these results indicate that both genetic parameters and expected responses to selection are environment-dependent, underscoring the need to model GxE explicitly to maintain prediction accuracy and realized gain across heterogeneous production environments.

To effectively model GxE, reaction norm (RN) models have emerged as a powerful tool, to describe genetic sensitivity across continuous environmental gradients and thereby improve the accuracy of selection under varying environmental conditions [21, 29]. The advent of genomics, particularly genome-wide association studies (GWASs), has enabled the dissection of complex trait architecture and more precise selection decisions [8, 30]. In Nellore cattle, GWAS has uncovered genomic regions associated with key production traits, including reproduction [31], growth [32], feed efficiency [4, 33], and carcass quality [34, 35]. Previous multi-trait GWAS identified signals on BTA5, 6, 14, and 16, accounting for up to ~ 9% of the genetic variance in HC30, while emphasizing the need for more robust methods due to strong environmental effects [31].

Integrating RN models with GWAS enables the mapping of genetic plasticity and environmental robustness, allowing for the identification of loci whose effects change across environmental gradients [36]. Consequently, this study aims to leverage the powerful combination of RN models with GWAS to: (1) identify environmentally sensitive SNPs associated with sexual precocity traits (SC365, HC30 and APM); and (2) pinpoint candidate genes and biological pathways potentially involved in the regulation of sexual precocity under heterogeneous environments.

## Materials and methods

No animal care committee approval was necessary for the purposes of this study, as all information required was obtained from existing databases.

### Phenotypic and pedigree data

The data used in this study were sourced from the Nellore Cattle Breeding Program (Programa Nelore Brazil), coordinated by the National Association of Breeders and Researchers (ANCP, Ribeirão Preto, São Paulo, Brazil; https://www.ancp.org.br/). The pedigree file includes information on approximately 1.5 million animals born between 1980 and 2023, distributed across 294 farms dedicated to the Nellore breed. The phenotypic data included records for three sexual precocity indicator traits: HC30, SC365, and APM.

The HC30 was assessed in Nellore heifers, which were exposed to reproduction between 12 and 14 months during their weaning year, over a 90-day breeding season to evaluate their ability to calve by 30 months. These heifers were managed on pasture under adequate nutritional support, receiving protein and mineral supplementation without feed restrictions. A score of 2 (success) was assigned to females that calved by 30 months, and 1 (failure) to those that calved after 30 months. The SC365 trait was measured using a scrotal tape at the point of maximum circumference (in cm) in young bulls and adjusted to 365 days of age.

The APM trait was evaluated through a combination of ultrasonographic evaluations and andrological examinations. Beginning at weaning (210 days), young bulls with a scrotal circumference of at least 19 cm underwent four testicular ultrasonography sessions over a 90-day period. Following these evaluations, clinical andrological exams and semen collections were performed. Puberty was confirmed based on the presence of ejaculates with progressive sperm motility (≥ 10%) and total sperm concentration ≥ 50 × 10⁶ sperm per ejaculate, following the criteria described by [37]. Ultrasonographic evaluations of the testicular parenchyma were performed using a 7.5 MHz linear probe, with images captured in the longitudinal-lateral plane. Based on these assessments, animals are classified as super-precocious (pubertal ≤ 14 months of age), precocious (pubertal between 14 and 17 months), or traditional (pubertal > 17 months) for practical proposes. A summary of the phenotypic records for each trait is shown in Table 1.

**Table 1:** Descriptive statistics for heifer early calving probability (HC30), scrotal circumference at 365 days of age (SC365), and age at puberty in males (APM) in Nellore cattle

Trait	Records	Minimum	Maximum	Mean ± SD	Number of CG
SC365 (cm)	274,292	12.10	34.20	22.01 ± 2.69	7,102
APM (months)	17,453	7.57	23.27	16.34 ± 3.54	209
HC30 (%)*	73,889	-	-	45.62	927

*CG* Contemporary group, *SD* Standard deviation

*Percentage of success for heifer early calving at 30 months

In order to characterize environmental variation, contemporary group (CG) effects for body weight adjusted to 455 days (W455) were used as an environmental indicator. The CGs for the evaluated traits were defined based on animals that were born on the same farm during the same year, within the same birth season (trimester), and within the same management group: pre-weaning groups (from birth to weaning) and post-weaning groups (from weaning to yearling or the age at which the trait was measured). Additionally, the sex effect was also considered in the CG for W455. The distribution of animals across environmental indicator classes and the corresponding mean phenotypic values for HC30, SC365 and APM are shown in Supplementary Figure S1. A descriptive summary of W455 for each trait is provided in the Supplementary Material S2 to illustrate the distribution of environmental conditions represented in this study. Observations falling more than 3.5 standard deviations below or above the CG mean were excluded for SC365 and APM. Furthermore, CG without phenotypic variation for HC30 (i.e., all animals showed the same response category; 1 or 2), were excluded from the analysis. Only CGs with at least five phenotypic records were kept for the analysis.

### Genomic information

The genomic dataset included 42,693 animals (21,083 females and 21,610 males), genotyped with the 53,492 SNP markers (GGP Indicus, Neogen, Lincoln, Nebraska, USA). Among the genotyped females, 9,965 had phenotypic records for HC30, and included 619 sires and 2,019 dams with progenies recorded for HC30. For the males, 19,392 had phenotypic data for SC365, and 5,401 for APM. A total of 1,428 and 333 sires had progenies phenotyped for SC365 and APM, respectively. Additionally, 790 genotyped males had a pedigree relationship of at least 0.25 with animals phenotyped for SC365, while 15,876 and 8480 had the same level of relatedness to animals phenotyped for APM and HC, respectively.

The genomic quality control (QC) was performed separately for male and female using the QCF90 software [38], removing sexual chromosomes, autosomal markers with a minor allele frequency (MAF) lower than 0.05, significant deviations from Hardy-Weinberg equilibrium (*p* ≤ 10⁻⁵), and call rates for markers and samples below 0.90. After QC, 42,693 genotyped animals (21,083 females and 21,610 males) and 42,424 SNP markers were retained for further analyses.

### Statistical modeling

#### Environmental gradients

Genetic sensitivity to environmental variation for sexual precocity indicators was evaluated using a two-step genomic RN model. Environmental gradient (EG) were derived from CG solutions based on best linear unbiased estimates (BLUE) for W455, which integrates management and environmental conditions experienced by the animals. Because production-environment differences shifting W455 also affect sexual precocity in heifers and young bulls [39], providing a biologically grounded proxy for environmental quality. The BLUE solutions were obtained from animal model via single-step genomic BLUP (ssGBLUP), as follows:

$$\:\mathbf{y}=\mathbf{X}\boldsymbol{\upbeta\:}+\mathbf{Z}\boldsymbol{\upalpha\:}+\boldsymbol{e},$$

where **y** is the vector of phenotypic information for W455, **β** is the vector of systematic effects of CG; **α** is the vector of additive genetic effects; **X** and **Z** are the incidence matrices that relate the fixed (**β**) and additive genetic effects ($\:\boldsymbol{\upalpha\:}$) to the phenotype, respectively; and **e** is a vector of residual effects. The random effects were assumed to be normally distributed: $\:\mathbf{a}\sim\mathrm{N}(0,\mathbf{H}{{\upsigma\:}}_{\mathrm{a}}^{2})$ and $\:\boldsymbol{e}=\:\mathrm{N}(0,\mathbf{I}{{\upsigma\:}}_{\mathrm{e}}^{2})$, where $\:{{\upsigma\:}}_{\mathrm{a}}^{2}$ and $\:{{\upsigma\:}}_{\mathrm{e}}^{2}$ are the additive genetic and residual variances, respectively; **I** is the identity matrix, and **H** is the matrix that combines pedigree and genomic relationships [40]. The **H** inverse matrix ($\:{\mathbf{H}}^{-1}$) was created as:

$$\:{\mathbf{H}}^{-1}\mathrm{=}{\left[\begin{array}{cc}{\boldsymbol{A}}_{11}^{-1}&\:{\boldsymbol{A}}_{12}^{-1}\\\:{\boldsymbol{A}}_{21}^{-1}&\:{\boldsymbol{A}}_{22}^{-1}\end{array}\right]}^{-1}\mathrm{+}\left[\begin{array}{cc}\mathrm{0}&\:\mathrm{0}\\\:\mathrm{0}&\:{\mathbf{G}}^{-1}\mathrm{-\:}{\mathbf{A}}_{22}^{-1}\end{array}\right]$$

where $\:{\mathbf{A}}^{-1}$ corresponds to the inverse of the pedigree-based relationship matrix considering three generations, $\:{\mathbf{A}}_{22}^{-1}$ represents the inverse of the pedigree-based relationship matrix for genotyped animals, and $\:{\mathbf{G}}^{-1}$ is the inverse of the genomic relationship matrix. The **G** matrix was estimated according to VanRaden [41], using the default parameters of the blupf90 family program [38]:

$$\:\mathbf{G}=\:\frac{\mathbf{Z}\mathbf{Z}\mathbf{{\prime\:}}}{2{\sum\:}_{\mathrm{i}=1}^{\mathrm{m}}{\mathrm{p}}_{\mathrm{i}}(1-{\mathrm{p}}_{\mathrm{i}})},$$

where **Z = (M – P)**, in which **M** is the SNP incidence matrix, with m columns (number of SNP markers) and n rows (number of genotyped animals), and **P** is a matrix derived from genotype coding and allele frequencies.

The EG levels were obtained by standardizing the BLUE solutions of CGs with a mean of 0 and a standard deviation (SD) of 1, with values ranging from − 3 to + 3 standard deviations. The ssGBLUP models were implemented using the blupf90 + software [38].

#### Reaction norms

To estimate genetic parameters for SC365, HC30, and APM across EG levels, a single-trait single-step genomic RN model (ssGRN) was applied:

$$\:{\mathbf{y}}_{\mathbf{i}\mathbf{j}}=\mathbf{X}\mathbf{b}+{\boldsymbol{\upomega\:}}_{\mathbf{f}}{\Phi}_{\mathbf{f}}\left({\mathbf{E}\mathbf{G}}_{\mathbf{j}}\right)+{\boldsymbol{\upalpha\:}}_{\mathbf{f}\mathbf{i}}{\Phi}_{\mathbf{f}}\left({\mathbf{E}\mathbf{G}}_{\mathbf{j}}\right)+{\mathbf{e}}_{\mathbf{i}\mathbf{j}},$$

where $\:{\mathbf{y}}_{\mathbf{i}\mathbf{j}}$ is the vector of phenotypic records for SC365, HC30, or APM of animal i at EG j; **b** is the vector of systematic CG effects; **X** is the incidence matrix; $\:{\boldsymbol{\upomega\:}}_{\mathbf{f}}$ is the f-th fixed regression coefficients for the intercept and slope on $\:{\boldsymbol{\Phi\:}}_{\mathbf{f}}\left({\mathbf{E}\mathbf{G}}_{\mathbf{j}}\right);\:{\boldsymbol{\Phi\:}}_{\mathbf{f}}\left({\mathbf{E}\mathbf{G}}_{\mathbf{j}}\right)$ is the vector containing the f-th Legendre orthogonal polynomials corresponding to EG j; $\:{\boldsymbol{\upalpha\:}}_{\mathbf{f}\mathbf{i}}$ is the vector of random regression coefficients for the additive genetic effects of intercept or slope corresponding to animal i at $\:{\mathrm{E}\mathrm{G}}_{\mathrm{j}}$; and $\:{\mathbf{e}}_{\mathbf{i}\mathbf{j}}$ is the vector of random residual effects. The additive genetic and residual effects were considered normally distributed: $\:\mathrm{a}\mathrm{=}\left\{{\mathrm{a}}_{\mathrm{j}}\right\}\sim\mathrm{N}\left(\mathrm{0,\:}\mathrm{H}\otimes\mathrm{K}\right)$ and $\:\mathrm{e}\mathrm{=}\left\{{\mathrm{e}}_{\mathrm{ij}}\right\}\sim\mathrm{N}(0,\mathbf{I}{\sigma\:}_{e}^{2}\mathrm{)},$ where $$\:\mathbf{K}=\left[\begin{array}{cc}{{\upsigma\:}}_{{\mathrm{a}}_{int}}^{2}&\:{{\upsigma\:}}_{{\mathrm{a}}_{int},{\mathrm{a}}_{slop}}\\\:{{\upsigma\:}}_{{\mathrm{a}}_{int},{\mathrm{a}}_{slop}}&\:{{\upsigma\:}}_{{\mathrm{a}}_{slop}}^{2}\end{array}\right]\:$$ is the genetic variance-covariance matrix for the intercept ($\:{\mathrm{a}}_{int}$**)** and slope ($\:{\mathrm{a}}_{slop}$) and ⊗ denotes the Kronecker product.

Although HC30 was originally recorded as a binary trait, the GWAS was performed under a linear framework. Therefore, variance components were estimated via Restricted Maximum Likelihood (REML), and GEBVs were obtained using BLUP, both implemented in the blupf90 + program [38]. To validate this approach, threshold (liability-scale) variance components were converted to the observed scale using the equation of [42] and compared with those estimated under the linear model. The similar magnitudes between both approaches suggest that the linear framework provides a reasonable approximation for HC30, justifying its use for the GWAS analysis (parameter comparison provided in the Supplementary Material S3). Variance components estimated in the initial run were subsequently fixed to predict GEBVs. For APM and SC365, the single-trait ssGRN models were implemented using Bayesian inference available in the gibbsf90 + software, part of the BLUPF90 family programs [38]. Each analysis consisted of a Markov Chain with 500,000 iterations. For APM, a burn-in of 200,000 iterations and a thinning interval of 5 were used, resulting in 60,000 independent samples for parameter estimation. For SC365, a burn-in of 100,000 iterations and a thinning interval of 5 were used, resulting in 80,000 samples for parameter estimation. Convergence was assessed using the Geweke [43] and Heidelberger & Welch [44] tests implemented in the Bayesian Output Analysis (BOA) R package [45]. After convergence, variance components were fixed to obtain the GEBVs, using BLUPF90+ [38].

### SNP effects and significance

The SNP effects for the intercept and slope were obtained using the postGSF90 software [46]. This tool enables the conversion of additive genomic random regression coefficients, represented by GEBVs for intercept and slope, into SNP solutions. The back-solving process was performed according to [47], as follows:

$$\:\hat{\mathbf{u}}_{\mathbf{c}}={\mathbf{M}}^{\mathbf{{\prime\:}}}{\left[\mathbf{M}{\mathbf{M}}^{\mathbf{{\prime\:}}}\right]}^{-1}\widehat{{\mathrm{a}}_{\mathbf{c}}},$$

where $\:\hat{\mathbf{u}}_{\mathbf{c}}$​ is the vector of SNP solutions for the c-th random regression coefficient (either intercept or slope), $\:\mathbf{M}$ is the matrix of centered genotypes (coded as 0, 1 and 2 for genotypes AA, AB, and BB, respectively), and $\:\widehat{{\mathrm{a}}_{\boldsymbol{c}}}$ is the vector of genomic breeding values for the RN coefficient obtained through ssGRN.

Approximate p-values for each SNP tested were obtained with the postGSF90 program based on the following equation [48]:

$$\:{\mathrm{p}}_{\mathrm{i}}=2(1-{\Phi\:}(\left|\frac{{\mathrm{u}}_{\mathrm{i}}}{\mathrm{s}\mathrm{d}\left({{\upalpha\:}}_{\mathrm{I}}\right)}\right|\left)\right),$$

where $\:{\mathrm{u}}_{\mathrm{i}}$ is the SNP effect estimate; sd is the standard deviation of the individual prediction error variance of the SNP effect estimate, following the approach proposed by [49]; and $\:{\Phi\:}$ is the standard normal cumulative distribution function. The p-values were obtained by back-solving the SNP effects from the GEBVs, using the postGSF90 program [46]. The Bonferroni correction method (alpha = 0.1) was applied to account for multiple testing, considering the number of independent chromosomal segments as [50], the average chromosome length, and the effective population size (*n* = 196 [51]), at the chromosome-wide level. This correction adjusts the significance thresholds of the SNPs accordingly, reducing the likelihood of false positives [52]. Given the highly polygenic nature of the traits analyzed, with many loci expected to have minor effects, a significance threshold of –log₁₀(p-value) > 5 (*p* < 10⁻⁵) was adopted for declaring SNPs as associated. Using a more stringent threshold would substantially increase the risk of Type II error, potentially masking biologically meaningful signals. Thus, the chosen threshold represents a compromise between controlling Type I error (false positives) and maintaining sufficient power to detect small-effect loci of biological interest. Given the 42,424 SNPs tested at this threshold, fewer than one false-positive association is expected under the global null hypothesis.

Next, the SNP solutions for the intercept and slope of each significant SNP 𝑘 ($\:{-log}_{10}\left(p-value\right)>5$) were combined into a vector **(**$$\:\hat{\boldsymbol{u}}_{\boldsymbol{k}}=\left[\begin{array}{c}\hat{\boldsymbol{u}}_{\boldsymbol{k},\boldsymbol{i}\boldsymbol{n}\boldsymbol{t}}\\\:\hat{\boldsymbol{u}}_{\boldsymbol{k},\boldsymbol{s}\boldsymbol{l}\boldsymbol{o}\boldsymbol{p}\boldsymbol{e}}\end{array}\right]$$), and then used to estimate the SNP effects across EG, the following equation:

$$\:{\mathbf{S}\mathbf{N}\mathbf{P}}_{\mathbf{k}}={\mathbf{T}\hat{\mathbf{u}}}_{\mathbf{k}},$$

where $\:{\boldsymbol{S}\boldsymbol{N}\boldsymbol{P}}_{\boldsymbol{k}}$​ is the vector of SNP effects for the ***k-th*** SNP across all EG values (ranging from − 3 to + 3 SD), and $\:\mathbf{T}$ is the matrix of evaluated Legendre orthogonal polynomials corresponding to each EG point. This approach allows the reconstruction of the SNP effect across the EG, capturing the dynamic contribution of each marker over environmental sensitivity.

### Gene annotation and functional analyses

Gene annotation was performed using the GALLO package [53] implemented in R (R Core Team, 2024), considering a genomic window of 100 kb upstream and downstream from each significant SNP. This step was based on the Ensembl database (https://useast.ensembl.org/info/data/ftp/index.html) and the ARS-UCD1.2 bovine genome assembly [54]. Positional candidate genes were then subjected to functional enrichment analysis using the gprofiler2 R package [55]. Enrichment was evaluated for the following Gene Ontology (GO) categories: Biological Processes (BP), Molecular Functions (MF), and Cellular Components (CC); as well as the Kyoto Encyclopedia of Genes and Genomes (KEGG) pathways. Given the highly polygenic architecture of the traits examined, it was not feasible to assign a single putative causal gene to each associated region. All genes whose genomic coordinates overlapped the ± 100 kb windows surrounding significant SNPs were therefore retained as positional candidates for enrichment analyses.

QTL annotation was performed using the same significant SNPs and a genomic window of ± 100 kb, using the GALLO R package [53], considering the ARS-UCD1.2 as the reference genome [54]. The QTL information was retrieved from the Animal QTL Database [56] and a hypergeometric-based significance test was applied to identify overlaps with trait-associated QTLs reported in cattle. Over-representation analysis of QTL categories was then performed to explore potential broader phenotypic associations of the candidate regions. QTL enrichment analysis was performed using GALLO, and significance was assessed based on adjusted p-values (adj.pval < 0.10).

## Results

### Genome-wide association study results

**Fig. 1:**
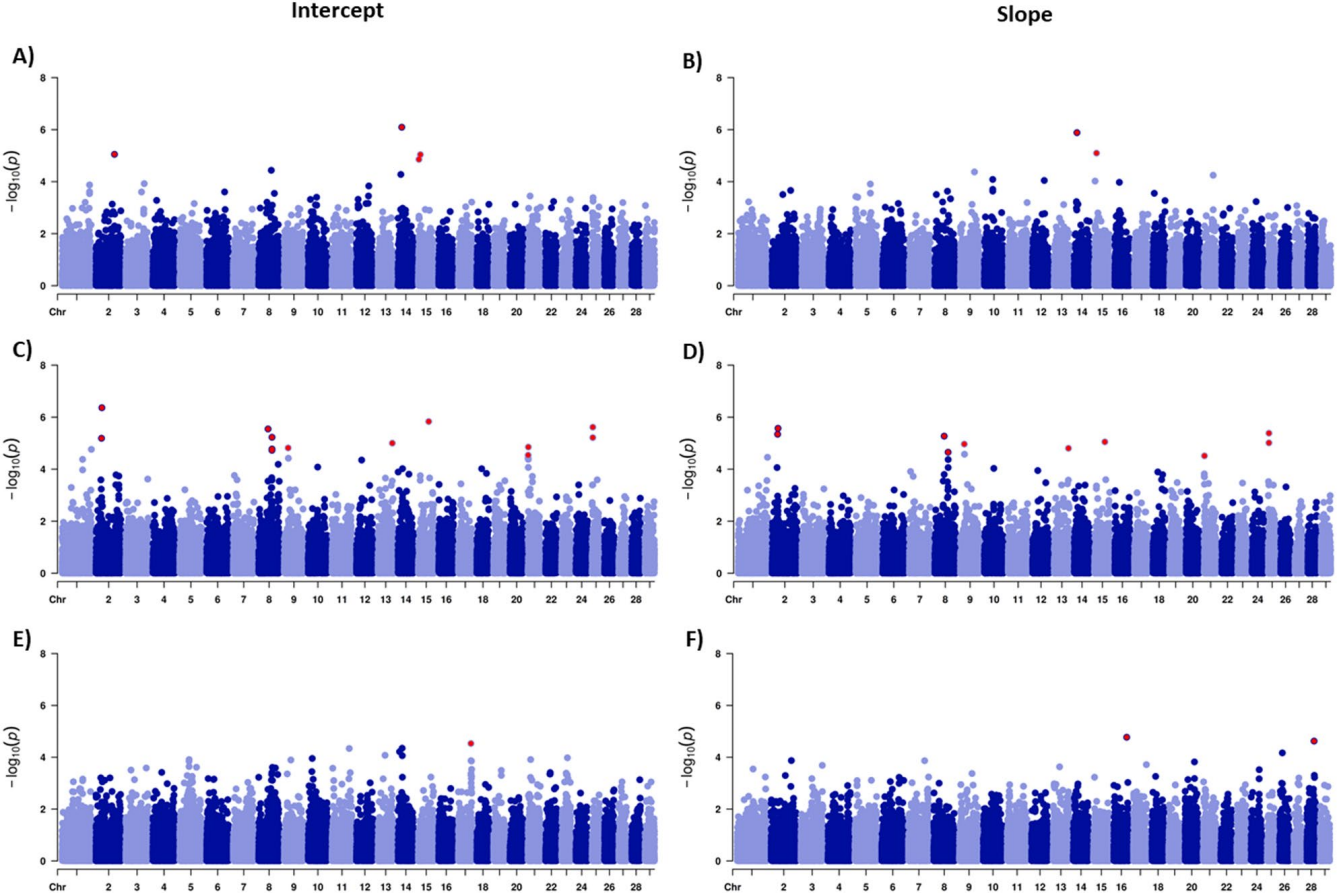
Manhattan plots for heifer early calving probability (HC30, **a** and **b**), scrotal circumference at 365 days of age (SC365, **c** and **d**), and age at puberty in males (APM, **e **and **f**) in Nellore cattle. The right panels (**a**, **c**, and **d**) represent the intercept, and the left panels (**b**, **d**, and **f**) the slope. Red dots indicate significant SNPs (*P*-value < 0.1) after multiple-test Bonferroni correction

**Fig. 2:**
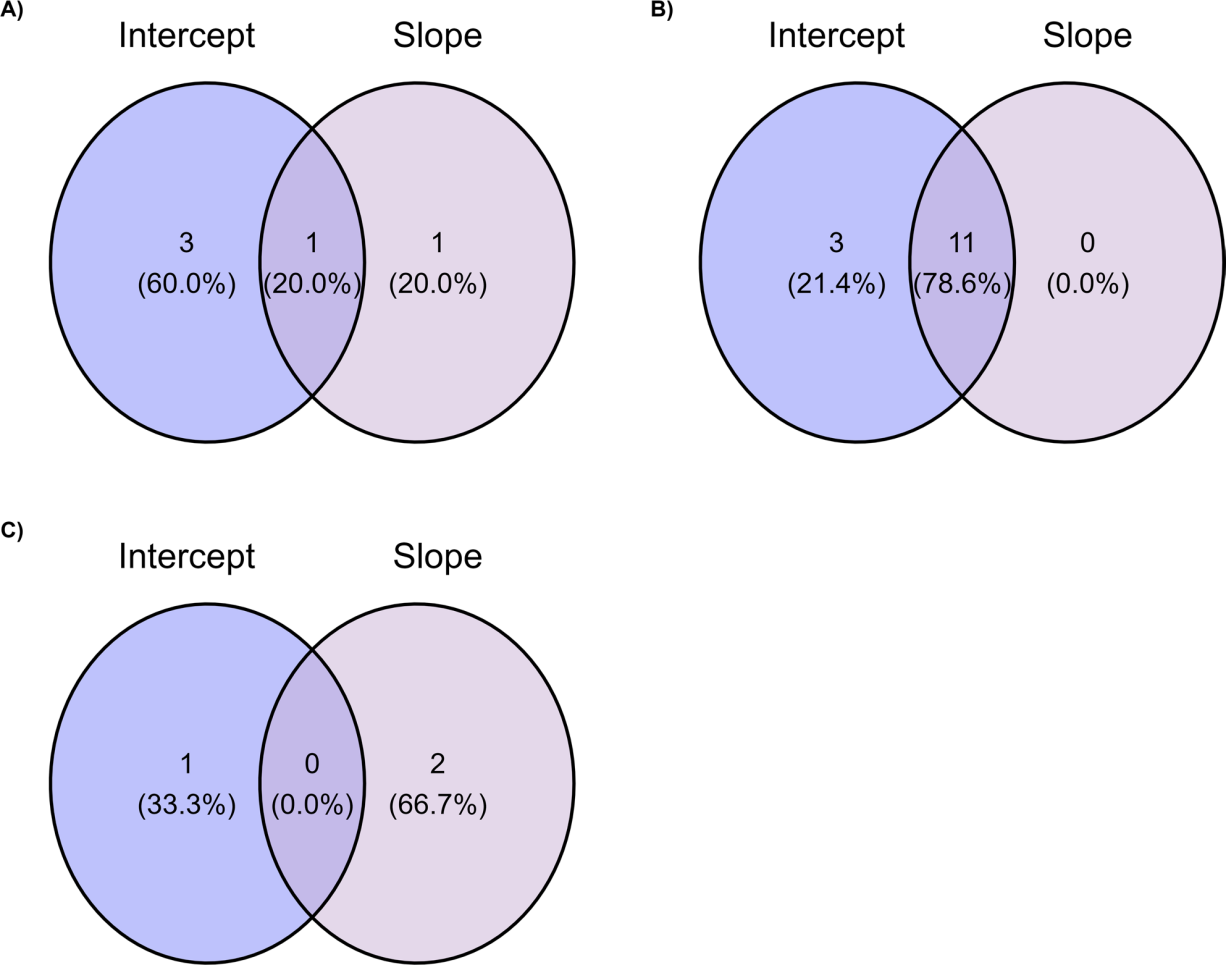
Venn diagram illustrating the overlap of significant SNPs between intercept and slope components identified for heifer early calving probability (HC30, **A**) and scrotal circumference at 365 days of age (SC365, **B**), and no overlap in age at puberty in males (APM, **C**) in Nellore cattle

Manhattan plots displaying GWAS results for intercept and slope of RN model for the evaluated traits are shown in Fig. 1. The genomic inflation factors (λ) were approximately 1.0 for all analyses, indicating appropriate control of population stratification. QQ-plots for intercept and slope components of HC30, SC365, and APM, showing the distribution of observed versus expected p-values are provided in Supplementary Figure S4. Venn diagrams (Fig. 2) show the distribution of significant SNPs between intercept and slope components for HC30, SC365, and APM. For HC30, a total of five significant SNPs were identified, with three associated exclusively with the intercept, one with the slope, and one shared between both components. For SC365, 14 SNPs were detected, of which three were unique to the intercept and 11 were common to both intercept and slope, with no slope-exclusive markers. For APM, three SNPs were identified, with one associated exclusively with the intercept, two with the slope, and no overlap between components. Some of the significant SNP markers identified were shared among RN parameters, contributing to the genetic correlations between intercept and slope. The genetic correlation between intercept and slope differed among traits, with values of 0.58 for HC30, 0.80 for SC365 and − 0.03 for APM.

**Fig. 3:**
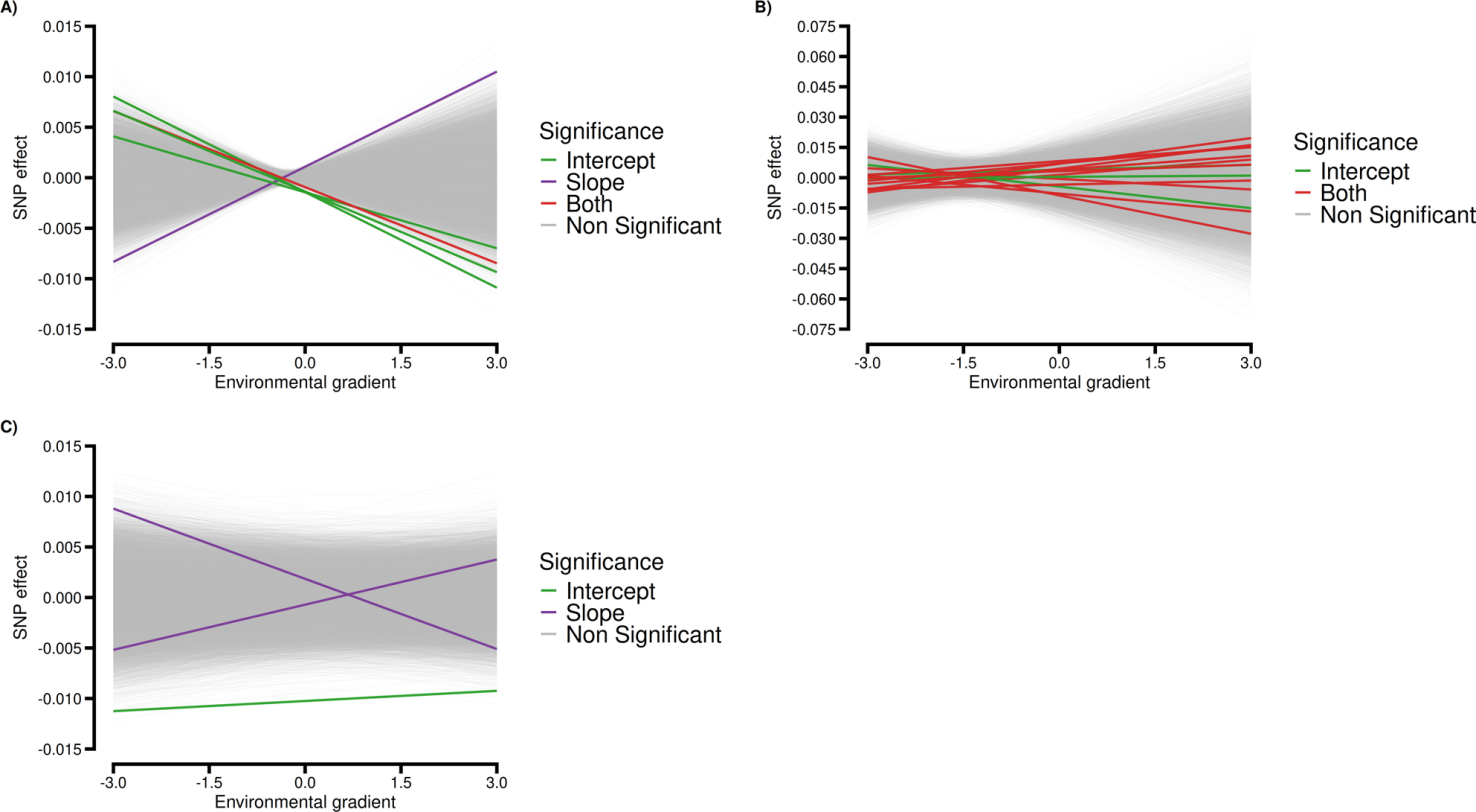
Variation in SNP effects along the environmental gradient for heifer early calving probability (HC30; *n* = 5 significant SNPs, **A**), scrotal circumference at 365 days of age (SC365; *n* = 14 significant SNPs, **B**), and age at puberty in males (APM; *n* = 3 significant SNPs, **C**) in Nellore cattle

Figure 3 shows the profiles of significant SNPs effects across the environmental gradient, providing insight into the environmental sensitivity of loci associated with each trait. Significant SNPs reveal trait-specific architectures of genetic plasticity (Fig. 3). For HC30, their effects change considerably across the environmental gradient, with the significant SNPs changing the sign effect when the EG becomes more favorable (Fig. 3A). For SC365, most loci combine genetic potential differences with environmental responsiveness (Fig. 3B). Slopes are consistent and produce expanding divergence of SNP effects toward environmental extremes. Thus, alleles affect both the mean expression of the trait and its reactivity to the environment. For APM, significant loci are classified as either slope-only or intercept-only types (Fig. 3C). Slope-only SNPs show opposing trends across the gradient, indicating a flip effect under different conditions (G×E). In contrast, intercept-only SNPs have a stable effect shift with little sensitivity to environmental changes.

### Candidate genes and enrichment analyses

Candidate genes located near significant SNPs for each trait are shown in Table 2, with results shown separately for the intercept and slope parameters. A total of 12 positional candidate genes were identified within the genomic regions associated with HC30, comprising seven protein-coding genes, one small nucleolar RNA (snoRNA), and four long non-coding RNAs (lncRNA). Notably, no genes were found to be associated with both intercept and slop for HC30 (Supplementary Table S5). For SC365, a substantially larger number of positional candidate genes was identified, with 98 genes located within the associated genomic regions. These included protein-coding genes, lncRNA, and small nucleolar RNAs (snoRNA). Interestingly, five genes (i.e., *GRB14*,* SLC9A8*,* SPATA2*,* MGRN1*, and *SEPTIN12*) were identified as significant for both intercept and slope components for SC365, suggesting their potential involvement in both baseline reproductive performance and environmental sensitivity (Supplementary Table S6). For APM, 12 positional candidate genes were identified within the associated genomic regions, comprising 6 protein-coding genes and 6 lncRNAs. Similar to HC30, no genes were identified as significant for both intercept and slop simultaneously (Supplementary Table S7).

**Table 2:** Candidate genes identified for intercept and slop of heifer early calving probability (HC30), scrotal circumference at 365 days of age (SC365), and age at puberty in males (APM) in Nellore cattle

Gene	Chromosome	SNP Position	Start - End Position	Gene Ensembl id
*HC30 - Intercept*				
* ERBB4*	2	100,036,095	99,222,774–100,507,400	ENSBTAG00000012647
* SNAI2*	14	19,972,663	19,941,415–19,945,240	ENSBTAG00000013227
*HC30 - Slope*				
* TRIB1*	14	14,769,483	14,779,050–14,787,206	ENSBTAG00000023179
* NSMCE2*	14	14,769,483	14,849,669–15,080,892	ENSBTAG00000009394
*SC365 - Intercept*				
* GRB14*	2	31,663,929	31,681,727–31,810,646	ENSBTAG00000019291
* SLC9A8*	13	77,958,705	77,899,068–77,973,805	ENSBTAG00000008969
* SPATA2*	13	77,958,705	77,982,558–77,993,066	ENSBTAG00000018063
* MGRN1*	25	3,865,404	3,755,133–3,793,240	ENSBTAG00000018999
* SEPTIN12*	25	3,865,404	3,856,459–3,868,939	ENSBTAG00000016330
*SC365 – Slope*				
* GRB14*	2	31,663,929	31,681,727–31,810,646	ENSBTAG00000019291
* SLC9A8*	13	77,958,705	77,899,068–77,973,805	ENSBTAG00000008969
* SPATA2*	13	77,958,705	77,982,558–77,993,066	ENSBTAG00000018063
* MGRN1*	25	3,865,404	3,755,133–3,793,240	ENSBTAG00000018999
* SEPTIN12*	25	3,865,404	3,856,459–3,868,939	ENSBTAG00000016330
*SC365 - Both*				
* GRB14*	2	31,663,929	31,681,727–31,810,646	ENSBTAG00000019291
* SLC9A8*	13	77,958,705	77,899,068–77,973,805	ENSBTAG00000008969
* SPATA2*	13	77,958,705	77,982,558–77,993,066	ENSBTAG00000018063
* MGRN1*	25	3,865,404	3,755,133–3,793,240	ENSBTAG00000018999
* SEPTIN12*	25	3,865,404	3,856,459–3,868,939	ENSBTAG00000016330
*APM - Intercept*				
* CHEK2*	17	68,042,761	67,975,026–68,012,641	ENSBTAG00000004956
* XBP1*	17	68,042,761	68,052,009–68,057,008	ENSBTAG00000005970
* TNIP3*	17	68,042,761	68,140,394–68,209,509	ENSBTAG00000047107

Significant enrichment results were obtained only for SC365 and HC30 (Table 3). The biological enrichment analysis (GO) for the gene set affecting the intercept for HC30 revealed terms related to growth-factor response (GO:0071364; GO:0070849), cell–cell junction organization/adhesion (GO:0002934; GO:0034330; GO:0033629), and cellular differentiation pathways (GO:0048863; GO:0048762; GO:0014033). For the slope component of HC30, enriched terms centered on immune/myeloid lineage regulation, including the negative regulation of LPS-mediated signaling (GO:0031665) and the positive/overall regulation of macrophage, neutrophil, and eosinophil differentiation (GO:0045651; GO:0045658; GO:0045643). The combined SC365 gene set (intercept and slope) was enriched for nuclear/condensate related cellular components nucleolus (GO:0005730), membraneless organelle and its intracellular subset (GO:0043228; GO:0043232), nuclear lumen (GO:0031981), and intracellular/organelle lumen (GO:0070013). The complete list of positional candidate genes is provided in the Supplementary Tables S5 to S7, for HC30, SC365, and APM, respectively. The complete lists of enriched GO terms for these traits are provided in the Supplementary Tables S8 and S9, for SC365 and HC30, respectively. In accordance with the mapping strategy described in the Material and Methods, the enriched GO terms are interpreted as functional themes shared by genes located in associated regions, not as evidence that each listed gene is causal or that a single gene explains the association at a given locus. Accordingly, the enrichment results are considered hypothesis-generating and provide a pathway-level framework to guide future functional studies aimed at identifying causal genes and mechanisms.

**Table 3:** Gene ontology terms for the genes annotated for heifer early calving probability (HC30) and scrotal circumference at 365 days of age (SC365) traits in Nellore cattle

Functional terms	*p*-value		Description of function
*HC30 - Intercept*			
GO:0071364	0.006		Cellular response to epidermal growth, factor stimulus
GO:0070849	0.006		Response to epidermal growth factor
GO:0002934	0.042		Desmosome organization
GO:0034330	0.049		Cell junction organization
GO:0033629	0.039		Negative regulation of cell adhesion, mediated by integrin
GO:2,000,209	0.046		Regulation of anoikis
GO:0048863	0.035		Stem cell differentiation
GO:0048762	0.035		Mesenchymal cell differentiation
GO:0014033	0.013		Neural crest cell differentiation
*HC30 - Slope*			
GO:0031665	0.024		Negative regulation of lipopolysaccharide-mediated signaling pathway
GO:0045651	0.024		Positive regulation of macrophage differentiation
GO:0045658	0.024		Regulation of neutrophil differentiation
GO:0045643	0.024		Regulation of eosinophil differentiation
*SC365 - Intercept and Slope*			
GO:0005730	6.9 × 10^− 06^		Nucleolus
GO:0043228	7.6 × 10^− 05^		Membraneless organelle
GO:0043232	7.5 × 10^− 05^		Intracellular, membraneless organelle
GO:0031981	0.005		Nuclear lumen
GO:0070013	0.007		Intracellular, organelle lumen

### QTL enrichment analyses

QTL enrichment identified significant overlaps between genomic regions associated with the intercept and slope of sexual precocity traits and known cattle QTLs. These overlaps covered reproductive and production categories for all traits evaluated (Table 4). The intercept for HC30 significantly overlap with calving ease QTLs on BTA15 (adjusted p-value = 0.068), while the slope component overlapped with non-return rate QTLs on BTA14 (adjusted p-value = 0.087) and calving ease QTLs on BTA15 (adjusted p-value = 0.087). For SC365, both intercept and slope significantly overlaps QTLs regions related to the calving ease on BTA21 (adjusted p-values = 1 × 10⁻³⁰ and 3.31 × 10⁻⁵, respectively), indicating strong genetic associations between scrotal circumference and calving performance. For APM, the intercept component showed a significant overlap QTLs associated with metabolic body weight on BTA17 (adjusted p-value = 2.78 × 10⁻¹⁷), suggesting potential pleiotropic effects between male sexual maturity and metabolic traits.

**Table 4:** QTL enrichment results identified for intercept and slop of heifer early calving probability (HC30), scrotal circumference at 365 days of age (SC365), and age at puberty in males (APM) in Nellore cattle

QTL	Chromosome	adj.pval	QTL_type
*HC30 - Intercept*			
Calving ease	15	0.068	Reproduction
*HC30 - Slope*			
Non-return rate	14	0.087	Reproduction
Calving ease	15	0.087	Reproduction
*SC365 - Intercept*			
Calving ease	21	1.0 × 10⁻³⁰	Reproduction
*SC365 - Slope*			
Calving ease	21	3.3 × 10⁻⁵	Reproduction
*APM - Intercept*			
Metabolic body weight	17	2.8 × 10⁻¹⁷	Production

QTL enrichment analysis was performed using GALLO, and significance was assessed based on adjusted p-values (adj.pval < 0.10)

## Discussion

This study investigated the genetic basis of GxE for sexual precocity in Nellore cattle combining RN models with ssGWAS. Compared to conventional GWAS, RN models enhance biological interpretability by identifying markers that alter expression across various environmental conditions. Its assumptions include linear environmental response, dependence on the quality of the environmental descriptor, and reduced precision at gradient extremes. Nonetheless, RN models provide results into GxE that conventional single-environment analyses cannot capture.

Using three indicators of sexual precocity (HC30, SC365, and APM), the analyses identified environmentally sensitive SNPs mapping candidate genes, and enriched pathways involved in sexual precocity. By examining both intercept and slope components, the results provide insights into genetic potential and environmental sensitivity, offering a comprehensive understanding of the genetic architecture underlying sexual precocity in Nellore cattle.

The sexual indicator traits examined represent different aspects of reproductive maturity and have varying degrees of environmental sensitivity. The SC365 is a trait commonly used as an indicator of fertility in Nellore bulls and serves as an indirect selection criterion for age at first calving in females, as it is easily measured, exhibits moderate to high heritability estimates [13–15], and is genetically correlated with female traits, such HC30, with correlation estimates ranging from 0.11 to 0.48 [12, 57]. More recently, HC30 and APM have gained attention as indicators of sexual precocity in Nellore cattle due to their moderate heritability and direct relevance to early reproductive performance [10, 11, 16]. At a cross-trait level, no overlap of significant SNPs or positional candidate genes was detected among HC30, SC365, and APM, despite the genetic correlations reported for these traits. This supports a highly polygenic architecture, in which correlations arise from many small-effect loci rather than from a few significant pleiotropic genes. The differential patterns of significant SNPs and candidate genes identified across these traits reflect their distinct biological mechanisms and environmental responsiveness. The SNP effect across the environments (Fig. 3) showed that its additive effect were relatively similar under less favorable environments but became more dispersed as conditions improved. This pattern suggests that favorable environments allow greater expression of genetic differences, making contrasts among SNP effects more evident. In contrast, restrictive environments dampen these differences and constrain the expression of SNP effects.

### Heifer early calving probability at 30 months (HC30)

For HC30, five significant SNPs were detected, three intercept-only, one slope-only, and one shared (Figs. 1 and 2). This pattern suggests that reproductive genetic potential and environmental sensitivity may be at least partially controlled by distinct genetic mechanisms in female sexual precocity. The *ERBB4* gene, identified as a candidate gene for the intercept (Table 2), encodes a tyrosine kinase receptor strongly associated with reproductive function [58]. Veikkolainen et al. [59] observed that *ERBB4* gene is essential for folliculogenesis and that its reduced expression can lead to reproductive dysfunction. Given that HC30 reflects sexual precocity in females, which relies on efficient folliculogenesis, the identification of *ERBB4* suggests a key role in establishing genetic potential for fertility in heifers. Another significant candidate gene for the intercept was *SNAI2* (Table 2), which acts as a transcriptional regulator in folliculogenesis and ovulation [60], suggesting its involvement in ovarian maturation and sexual precocity. Supporting this, Meng et al. [61] identified *SNAI2* as a regulatory factor in the uterine cervix and early gestation maintenance in sheep, reinforcing its participation in critical stages of the reproductive cycle, from ovarian function to embryo implantation.

Among the candidate genes identified for the slope (Table 2), *TRIB1* and *NSMCE2* are notable for their reported roles in cell stress and reproductive processes. The *TRIB1* gene has been associated with granulosa and theca cell function, ovulation, and steroidogenesis, as well as lipid metabolism and oxidative stress regulation [62–64]. These pleiotropic roles are compatible with a scenario in which variants near *TRIB1* influence the sensitivity of female fertility to metabolic and environmental challenges. The *NSMCE2* gene participates in DNA repair and resolution of topological stress during replication [65] and has been associated with adaptive and productive traits in domestic rabbits [66]. Its association with the slope component therefore supports the idea that cellular stress response pathways may modulate the environmental sensitivity of HC30. However, in both cases, our results are limited to statistical associations, and further functional work is needed to clarify whether these loci directly affect sexual precocity or tag nearby causal variants.

The functional distinction between genes identified for the intercept and slope reflects complementary biological mechanisms underlying sexual precocity in Nellore heifers (Table 3). Genes associated with the intercept were primarily enriched for growth factor signaling. In contrast, genes identified for slope were enriched for immune and inflammatory regulation, as well as cellular resilience pathways (Supplementary Table S9). While intercept genes appear closely related to genetic reproductive potential, slope-associated genes likely act as modulators under environmental or metabolic stress. These results suggest a mechanistic link between stress adaptation and female reproductive performance.

Reproduction in females requires tightly regulated inflammatory processes to support ovulation and implantation, whereas prolonged immune activation can impair ovarian function and embryonic survival [67, 68]. Genes that help the body to stop inflammation and protect cells against oxidative stress quickly can better support oocyte quality, placental development, and pregnancy maintenance, even under nutritional or environmental stress [69, 70]. On the other hand, alleles that cause prolonged or inefficient immune responses may reduce the resources available for reproduction, leading to delayed return to estrus and higher risk of embryonic loss [71]. This distinction may help explain why some heifers achieve superior reproductive performance when confronted with challenges such as nutritional limitations, climate variation, or health stressors. In line with this, the observed enrichments suggest that the ability to cope with immune and cellular stress is a key factor underlying variation in reproductive resilience, with direct implications for fertility and long-term productivity in cattle populations.

QTL enrichment analyses for intercept and slope genes revealed overlaps with regions previously associated with calving ease, and non-return rate (Table 4). Notably, the presence of calving ease is highly consistent with HC30, as both traits capture the ability of heifers to reproduce early, and they are also genetically correlated [12, 20]. Interestingly, non-return rate appeared exclusively among QTLs associated with slope, reinforcing the idea that this trait, which measures return to estrus after insemination, is strongly modulated by environmental effects.

### Scrotal circumference at 365 days (SC365)

The SC365 exhibited the most significant genetic markers (n. 14 SNPs), of which three were uniquely associated with the intercept and 11 were shared between the intercept and slope of the RN model (Figs. 1 and 2). This architecture contrasts with the other traits, indicating that SC365 may be influenced by relatively complex genetic mechanisms with considerable overlap between genetic potential (intercept) and environmental sensitivity (slope). Among the candidate genes significantly associated with the intercept (Table 2), two genes *MAGEL2* and *NDN* deserve to be highlighted. Both genes belong to the Prader-Willi/Angelman cluster, a conserved imprinted genomic region related to paternal expression that has been described in humans and cattle [72]. In mammals, these genes are involved in the neuroendocrine control of reproduction and fertility.

The *MAGEL2* gene influences circadian and hypothalamic regulation, and its absence in mice results in delayed puberty, reduced testosterone, and progressive infertility [73]. Similarly, *NDN* is essential for the survival and migration of GnRH neurons, thus controlling the onset of puberty and reproductive competence [74]. Overall, the detection of *MAGEL2* and *NDN* at the intercept level suggests that neuroendocrine regulation of puberty plays a significant role in defining the baseline genetic potential of SC365. Therefore, as this trait reflects testicular growth and reproductive capacity, these findings reinforce the role of hypothalamic pituitary signaling pathways in shaping male fertility in Nellore cattle.

An interesting aspect of SC365 is that all genes associated with environmental sensitivity (slope) were also detected for baseline potential (intercept), suggesting that the genetic architecture of SC365 is largely shared between these components. This finding is consistent with the literature, which describes SC365 as a robust trait in GxE studies. For instance [75], reported high genetic correlations for SC365 between environments, classifying animals as minimally sensitive. Similarly [76], found comparable results, reinforcing the robustness of SC365. Therefore, the absence of slope-exclusive signals likely reflects that the same genetic determinants underlying basal testicular development also control the limited environmental sensitivity of this trait.

The candidate gene *GRB14* (growth factor receptor-bound protein 14), identified as associated with both intercept and slope levels for SC365, is linked to insulin/IGF signaling pathways and has been detected in granulosa and theca cells during follicular growth in cattle [77, 78]. further highlighted *GRB14*’s role as a regulator of the balance between cellular growth and maturation. Given that SC365 reflects male reproductive performance by indicating testicular capacity for sperm and hormones production, these findings are compatible with a role of *GRB14* in sexual precocity through the regulation of cellular growth and differentiation. Interestingly, *GRB14* has also been associated with reproductive cells in avian species [79]. In Nellore cattle [23], identified *GRB14* through GWAS on SC365 and suggested that this gene influences not only male reproductive traits but also female fertility.

Another significant gene at both intercept and slope levels was *SLC9A8*, also known as the Na+/H + exchanger 8 (NHE8), which plays a role in cellular signaling, proliferation, and differentiation. In humans, it is expressed in Leydig and germ cells, where it is critical for sperm physiology [80, 81]. Studies in mice have demonstrated that *SLC9A8* knockout leads to defective acrosome formation and reduced testosterone production, resulting from lower luteinizing hormone receptor expression, ultimately contributing to male infertility [80]. These findings are consistent with *SLC9A8* contributing to spermatogenesis and testicular development.

The *SPATA2* gene was also significant for both the intercept and slope, and it plays a direct role in spermatogenesis and sperm quality, with its absence reducing male fertility [82]. *SPATA2* expression has been detected in mice and in different fish species [82–84]. Furthermore [85], demonstrated that *SPATA2* is involved in inflammation through TNF/NF–κB dependent signaling, suggesting a role in testicular resilience under environmental stressors. *MGRN1*, also detected for intercept and slope, has been implicated in bull fertility in other species and is directly involved in spermatogenesis [86]. *SEPTIN12*, another strong candidate at both intercept and slope for SC365, is well known for its importance in male fertility. Homozygous *SEPTIN12* knockout mice exhibit reduced sperm counts, abnormal morphology, and fertilization failure [87]. In humans, *SEPTIN12* variants have also been linked to sperm defects [88], confirming its essential role in maintaining sperm structural integrity.

The candidate genes were enriched in cellular processes related to nuclear function, membraneless organelles, and intracellular lumen organization (Table 3). Overall, the functional profile of the significant genes in SC365 indicates that the trait is driven primarily by nuclear and cellular processes, results that are consistent with the robust nature of this phenotype. Furthermore, QTL enrichment analysis revealed a substantial overlap with regions previously associated with calving ease (Table 4), a biologically coherent finding considering the genetic correlation between SC365 and female fertility traits [10, 19, 89]. Interestingly, calving ease enrichment was consistent for both intercept and slope, reinforcing the interpretation that the same genomic regions influence both and the limited environmental sensitivity of SC365.

### Age at puberty in males (APM)

For APM, three significant SNPs were identified, one for intercept baseline testis development and two for slope, with no overlap between them (Figs. 1 and 2). This pattern suggests that genetic potential for APM and environmental sensitivity are controlled by distinct genetic mechanism. The intercept-level signal maps to *CHEK2*, a checkpoint kinase central to DNA-damage sensing and cell-cycle control, provide a plausible mechanistic link to germ-cell and somatic support-cell proliferation during testis maturation [90]. While direct livestock evidence is lacking, human data associate *CHEK2* variation with reproductive phenotypes, including polycystic ovary syndrome and primary ovarian insufficiency, underscoring its role in gonadal function and. *CHEK2* has also been described as a sensor of metabolic and replicative stress in woman, ensuring genome stability under high proliferative demand [91]. Considering that testicular development and spermatogenesis are characterized by intense cell division and high metabolic activity, variation in *CHEK2* may contribute to differences in the onset of puberty in Nellore bulls, although further validation is required to confirm this hypothesis.

The candidate gene *XBP1*, identified for the intercept of RN model for APM, encodes a transcription factor that acts as a central regulator of the unfolded protein response during endoplasmic reticulum stress, thereby ensuring cellular homeostasis under high proliferative demand [92]. In goats [93], reported that overexpression of *XBP1* enhanced the proliferation and antioxidant capacity of spermatogonial stem cells, whereas its knockdown impaired growth, indicating that it is critical to protect germ cells from inflammatory stress [94]. emphasized that dysregulation of *XBP1* compromises reproductive performance by disrupting the balance between stress adaptation and cell survival in both male and female systems. These results suggest that *XBP1* may influence APM by modulating testicular development and the ability of germ cells to withstand metabolic stress, thereby affecting the initiation of spermatogenesis and sexual maturation. The *TNIP3* gene, also detected at the intercept level for APM, has previously been reported in Nelore cattle and associated with immune response and inflammation [95].

Notably, no protein-coding candidate genes were detected exclusively for the slope component in APM. This may be related to several factors. First, APM is a relatively novel trait that still requires further investigation. Another possibility is that baseline genetic effects dominate the onset of male puberty, making environmental effects more subtle. In line with this hypothesis, no significant GO terms were observed for APM (Table 3), which may indicate that no specific biological pathways are strongly concentrated in modulating this trait. QTL enrichment for APM at the intercept level revealed strong overlap with metabolic body weight (Table 4). This pattern is biologically coherent with the nature of APM, a trait that depends on reaching a minimum somatic and endocrine maturity. Metabolic body weight is a robust indicator of body size, energy availability, and overall growth status, which are key physiological factors for the activation of spermatogenesis. In contrast, slope enrichment was weak and diffuse, consistent with the limited environmental sensitivity observed for this trait.

Overall, the three indicators of sexual precocity differed in their sensitivity to environmental factors. For instance, HC30 exhibited the most substantial evidence of GxE, with slope-associated genes associated with immune and metabolic stress responses, highlighting its dependence on environmental conditions. In contrast, SC365 appeared to be the most robust trait, as nearly all significant loci were shared between intercept and slope, suggesting limited environmental modulation.

### Implications and future directions

The integration of RN models with ssGWAS has revealed distinct genetic architectures underlying sexual precocity traits in Nellore cattle, with important implications for breeding strategies and our understanding of reproductive biology. The differential genetic architectures identified suggest that trait-specific selection strategies should be implemented in breeding programs. The distinct genetic control of genetic potential (intercept) and environmental sensitivity (slope) identified for HC30 suggests that these components can be independently targeted in breeding programs. Moreover, the identification of genes like *TRIB1*, which connects metabolic pathways with reproductive function, suggests that selection for animal robustness in female fertility should consider metabolic efficiency to thrive under stressful environmental conditions.

For SC365, the shared genetic architecture between genetic potential and environmental sensitivity presents both opportunities and challenges. The overlap of genes across intercept and slope components underscores the robustness of this trait, implying that selection for improved SC365 may simultaneously enhance intrinsic reproductive capacity and resilience to environmental variation. This overlap can also be explained by the high genetic correlation between the intercept and slope (0.81), as it is expected that the stronger the relationship between the intercept and slope, the more likely they are to share significant markers. This makes SC365 particularly valuable as a selection criterion in variable production environments. The identification of genes like *GRB14*,* SLC9A8*,* SPATA2*,* MGRN1*, and *SEPTIN12* as pleiotropic regulators provides specific genomic targets for enhancing male reproductive performance across diverse environmental conditions. The limited environmental sensitivity observed for APM, combined with its strong association with metabolic body weight, suggests that selection strategies should focus primarily on baseline genetic potential while ensuring adequate nutritional management to support somatic growth and endocrine maturation. Furthermore, this trait exhibited a very low genetic correlation (-0.03), indicating a near-null relationship between the intercept and slope, which justifies the low environmental sensitivity observed.

The practical applications of these findings support the development of genomic selection indices that weight genetic potential and environmental sensitivity according to production system. In stable, high-input systems, emphasis could be placed on genetic potential, while in variable or challenging environments, greater weight could be given to environmental robustness markers. Future research priorities should focus on functional validation studies to confirm the roles of the candidate genes identified in this study. Experimental approaches using targeted expression studies are needed to dissect the causal mechanisms by which these loci influence reproductive development in Nellore cattle. Multi-omics integration, particularly transcriptomic and metabolomic data, will further clarify the pathways connecting metabolism, stress response, and reproduction. Expanding environmental descriptors beyond the W455-based framework to include climatic variables and nutritional challenges will enable a more comprehensive characterization of GxE interactions. Finally, exploring epigenetic mechanisms offers an additional layer of insight into the plasticity of sexual precocity, providing a deeper understanding of how environmental sensitivity is regulated at the molecular level.

### Limitations

Despite providing novel insights into the genetic and environmental components of sexual precocity, this study has several limitations. First, ssGWAS based on RN coefficients identifies statistical associations rather than causation, and the significant SNPs detected here may be in linkage disequilibrium with causal variants. Functional validation is required to confirm the biological roles of highlighted candidate genes. Second, predictions at the extremes of the environmental gradient may exhibit reduced precision, a known characteristic of RRM based on Legendre orthogonal polynomials, even though the dataset provided good connectedness across environments. Third, the enrichment analysis may overrepresent pathways, as all genes within associated windows were included, which reflects biological themes rather than unique causal genes.

## Conclusions

This study provides new understandings into the genetic architecture of sexual precocity traits in Nellore cattle by integrating RN models with GWAS. For HC30, genes associated with the intercept were linked to folliculogenesis and ovarian development, while those associated with the slope reflected metabolic regulation, immune responses, and cellular stress resilience. SC365 revealed a contrasting pattern, with most significant genes shared between intercept and slope, suggesting that the same genomic determinants contribute to both baseline testis development and limited environmental sensitivity. APM presented only intercept-associated candidate genes related to DNA repair, endoplasmic reticulum stress, and immune response, with no slope-exclusive protein-coding genes or enriched biological processes detected. Together, these findings underscore the importance of environmentally sensitive loci and pathways in influencing reproductive function, providing a more comprehensive understanding of how baseline genetic potential and environmental sensitivity contribute to sexual precocity in Nellore cattle.

## Supplementary Information

Supplementary Material 1.

## Data Availability

The phenotypic and genotypic information is available for academic use from the authors upon reasonable request (contacting the National Association of Breeders and Researchers (ANCP, Ribeirão Preto, SP, Brazil; email: [transf.tecnologia@ancp.org.br](mailto: transf.tecnologia@ancp.org.br) ).
