# Supplementary material for: Integrating reaction norm models and genome-wide association analyses to reveal the genetic architecture and environmental sensitivity of sexual precocity in Nellore cattle
Source: BMC Genomics. 2026 Jan 21;27:200. doi: 10.1186/s12864-026-12547-8 (PMC12908385; doi:10.1186/s12864-026-12547-8)
Supplement: Supplementary file 1 — Supplementary Material 1. [file 12864_2026_12547_MOESM1_ESM.docx]

**Integrating reaction norm models and genome-wide association analyses to reveal the genetic architecture and environmental sensitivity of sexual precocity in Nellore cattle**

Eduarda da Silva Oliveira*¹^,^², Hinayah Rojas de Oliveira^2^, Lúcio Flávio Macêdo Mota¹^,^², Henrique Alberto Mulim², Milena Aparecida Ferreira Campos²^,^³, João Barbosa da Silva Neto¹^,^², Fernando Baldi^4^

**SUPPLEMENTARY MATERIAL**

**
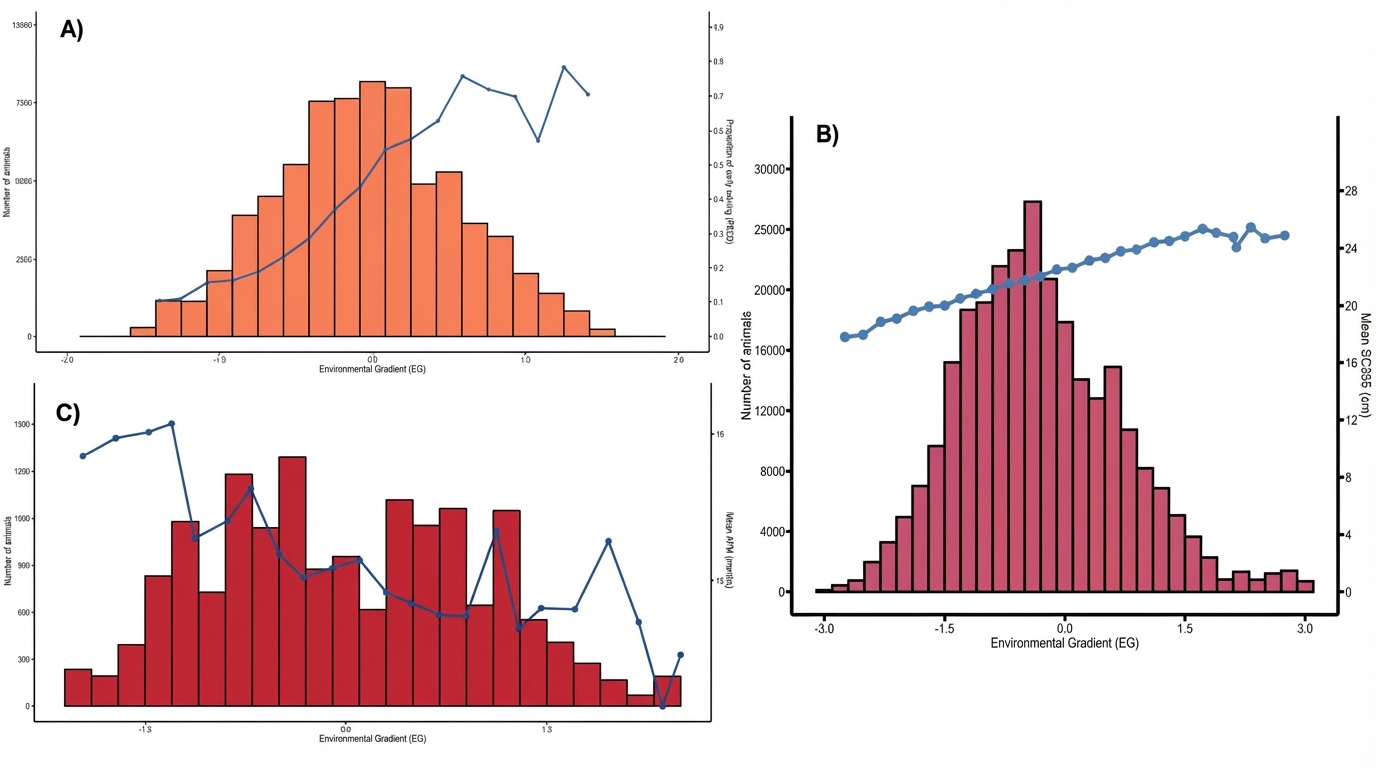
**

S1. Distribution of animals and mean sexual precocity indicators across the environmental gradient (EG) for heifer early calving probability (HC30, A), scrotal circumference at 365 days of age (SC365, B), and age at puberty in males (APM, C) in Nellore cattle. Bars represent the number of animals in each EG class, and the blue line indicates the mean value (or proportion, for HC30) of the respective trait within each class.

S2. Descriptive statistics of W455 across HC30, SC365 and APM traits in Nellore cattle.

| **Trait** | **Records** | **Minimum** | **Maximum** | **Mean** ± **SD** |
| --- | --- | --- | --- | --- |
| **HC30** | 73,889 | 155 | 596 | 305.93 ± 43.62 |
| **SC365** | 274,292 | 151 | 599 | 334.66 ± 62.37 |
| **APM** | 17,453 | 171 | 595 | 366.46 ± 59.7 |

HC30: heifer early calving probability at 30 months, SC365: scrotal circumference at 365 days, APM: age at puberty in males, SD: standard deviation.

S3. Comparison of variance components (σ²) and heritabilities (h²) for HC30 in Nellore cattle under threshold and linear models.

| **Component** | **σ² Liability (HPD 95%)** | **σ² Observed (HPD 95%)** | **h² Liability (HPD 95%)** | **h² Observed (HPD 95%)** | **σ² BLUP (CI)** | **h² BLUP (CI)** |
| --- | --- | --- | --- | --- | --- | --- |
| **Intercept** | 0.286 (0.22780;0.34990) | 0.035 (0.029;0.040) | 0.221 (0.187;0.256) | 0.140 (0.118;0.162) | 0.016 (0.013;0.019) | 0.104 (0.088;0.121) |
| **Slope** | 1.166 (0.93530;1.4140) | 0.084 (0.076;0,091) | 0.537 (0.486;0.581) | 0.340 (0.308;0.368) | 0.015 (0.013;0.019) | 0.098 (0.088; 0.121) |
| **Residual** | 1.005 (0.99050;1.0190) | 0.21 (0.208;0.219) | - | - | 0.137 (0.135;0.138) | - |

HC30: heifer early calving probability at 30 months; HPD: Highest Posterior Density interval; CI: confidence interval


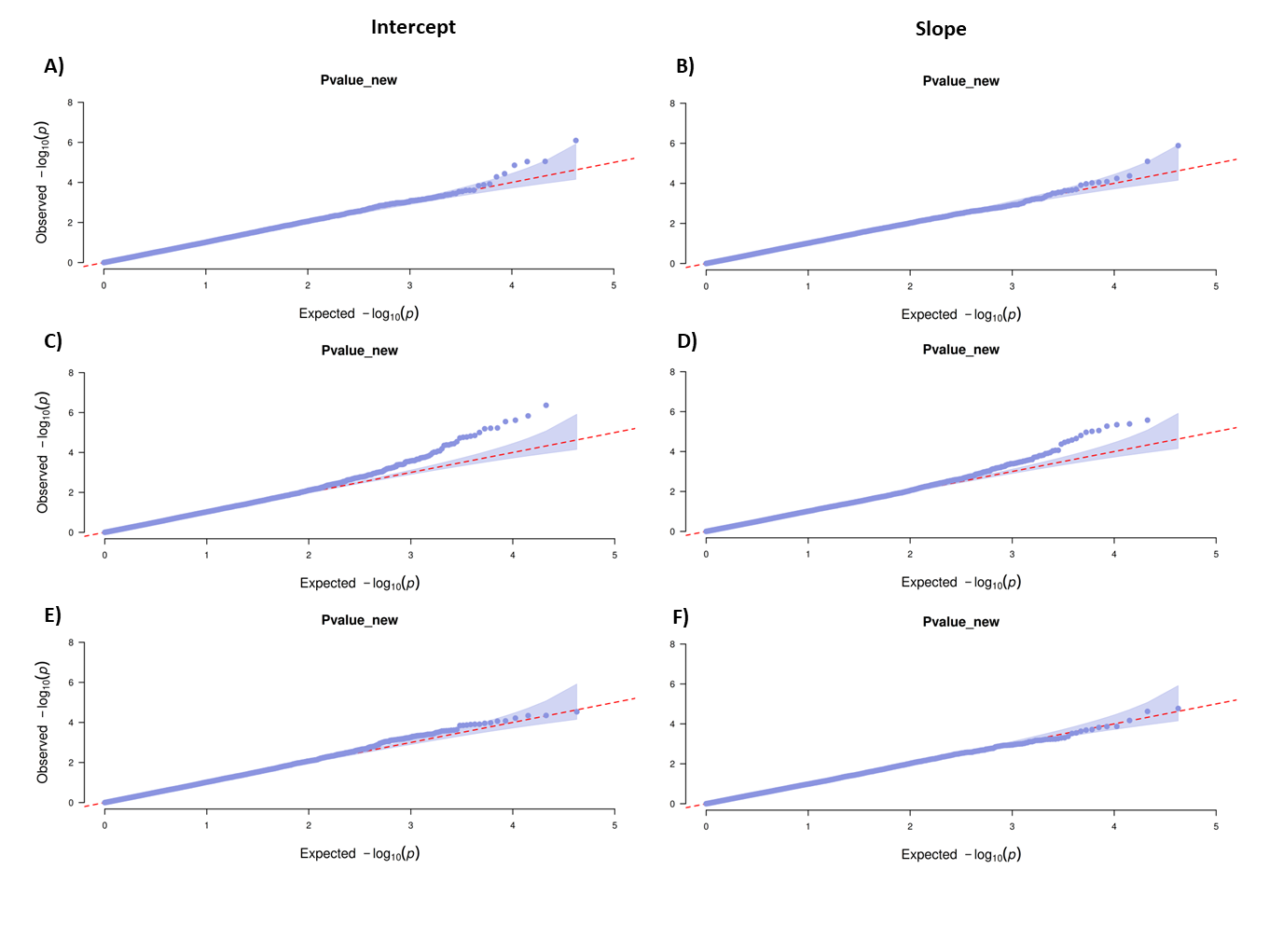


S4. Quantile-quantile (QQ) plots from the heifer early calving probability at 30 months (HC30, A and B), scrotal circumference at 365 days (SC365, C and D) and age at puberty in males (APM, E and F) with the lambda correction for 1.

S5. Positional candidate genes located near the regions identified for HC30 in Nellore cattle.

| **Gene** | **Chromosome** | **Position** | **Start - End Position** | **gene_id** | **gene_biotype** |
| --- | --- | --- | --- | --- | --- |
| **HC30 - Intercept** | |  |  |  |  |
| ***ERBB4*** | 2 | 100036095 | 99222774 – 100507400 | ENSBTAG00000012647 | protein_coding |
|  | 2 | 100036095 | 99976867 - 99976958 | ENSBTAG00000044975 | snoRNA |
| ***SNAI2*** | 14 | 19972663 | 19941415 - 19945240 | ENSBTAG00000013227 | protein_coding |
| ***PPDPFL*** | 14 | 19972663 | 19981881 - 19990059 | ENSBTAG00000038286 | protein_coding |
| ***CASP1*** | 15 | 3196766 | 3213132 - 3248824 | ENSBTAG00000050239 | protein_coding |
| ***CASP4*** | 15 | 3196766 | 3249849 - 3269687 | ENSBTAG00000020884 | protein_coding |
| **HC30 - Slope** |  |  |  |  |  |
| ***TRIB1*** | 14 | 14769483 | 14779050 - 14787206 | ENSBTAG00000023179 | protein_coding |
| ***NSMCE2*** | 14 | 14769483 | 14849669 - 15080892 | ENSBTAG00000009394 | protein_coding |
|  | 14 | 14769483 | 14787645 - 14789050 | ENSBTAG00000068933 | lncRNA |
|  | 14 | 14769483 | 14847096 - 14849364 | ENSBTAG00000060842 | lncRNA |
|  | 14 | 14769483 | 14792470 - 14802513 | ENSBTAG00000065404 | lncRNA |
|  | 14 | 14769483 | 14800117 - 14804502 | ENSBTAG00000066195 | lncRNA |

HC30: heifer early calving probability at 30 months

S6. Positional candidate genes located near the regions identified for SC365 in Nellore cattle.

| **Gene** | **Chromosome** | **Position** | **Start - End Position** | **gene_id** | **gene_biotype** |
| --- | --- | --- | --- | --- | --- |
| **SC365 - Intercept** | |  |  |  |  |
| ***COBLL1*** | 2 | 31663929 | 31429939 - 31597839 | ENSBTAG00000005501 | protein_coding |
| ***GRB14*** | 2 | 31663929 | 31681727 - 31810646 | ENSBTAG00000019291 | protein_coding |
|  | 2 | 31663929 | 31630290 - 31633063 | ENSBTAG00000077981 | lncRNA |
|  | 2 | 31663929 | 31641858 - 31681637 | ENSBTAG00000078676 | lncRNA |
|  | 2 | 31663929 | 31642351 - 31645531 | ENSBTAG00000071435 | lncRNA |
|  | 2 | 31663929 | 31660845 - 31664971 | ENSBTAG00000078032 | lncRNA |
| ***XIRP2*** | 2 | 29725662 | 28988612 - 29679060 | ENSBTAG00000017743 | protein_coding |
|  | 2 | 29725662 | 29713091 - 29714941 | ENSBTAG00000078998 | lncRNA |
| ***NEFM*** | 8 | 72120137 | 72181225 - 72186744 | ENSBTAG00000039530 | protein_coding |
| ***NEFL*** | 8 | 72120137 | 72213265 - 72217537 | ENSBTAG00000021949 | protein_coding |
|  | 8 | 71848112 | 71934349 - 71940741 | ENSBTAG00000077441 | lncRNA |
|  | 8 | 72120137 | 72176716 - 72178006 | ENSBTAG00000056739 | lncRNA |
|  | 8 | 72120137 | 72176720 - 72180383 | ENSBTAG00000077653 | lncRNA |
|  | 8 | 72120137 | 72205614 - 72209218 | ENSBTAG00000066147 | lncRNA |
|  | 8 | 72151421 | 72223223 - 72224668 | ENSBTAG00000071900 | lncRNA |
| ***RORB*** | 8 | 50615899 | 50700722 - 50763952 | ENSBTAG00000021699 | protein_coding |
|  | 8 | 50615899 | 50667933 - 50668002 | ENSBTAG00000042911 | snoRNA |
|  | 9 | 21153630 | 21142816 - 21150553 | ENSBTAG00000070448 | lncRNA |
|  | 9 | 21153630 | 21154216 - 21155355 | ENSBTAG00000075143 | lncRNA |
|  | 9 | 21153630 | 21220877 - 21232768 | ENSBTAG00000077273 | lncRNA |
|  | 9 | 21153630 | 21232855 - 21257112 | ENSBTAG00000057943 | lncRNA |
| ***SLC9A8*** | 13 | 77958705 | 77899068 - 77973805 | ENSBTAG00000008969 | protein_coding |
| ***SPATA2*** | 13 | 77958705 | 77982558 - 77993066 | ENSBTAG00000018063 | protein_coding |
| ***RNF114*** | 13 | 77958705 | 77993252 - 78017909 | ENSBTAG00000027317 | protein_coding |
| ***SNAI1*** | 13 | 77958705 | 78036441 - 78042568 | ENSBTAG00000014554 | protein_coding |
| ***SNORA70*** | 13 | 77958705 | 78012097 - 78012227 | ENSBTAG00000044591 | snoRNA |
| ***ACER3*** | 15 | 56246427 | 56246786 - 56447013 | ENSBTAG00000017218 | protein_coding |
| ***TSKU*** | 15 | 56246427 | 56178753 - 56204248 | ENSBTAG00000058190 | protein_coding |
|  | 15 | 56246427 | 56174504 - 56176334 | ENSBTAG00000074927 | lncRNA |
| ***MAGEL2*** | 21 | 1278161 | 1204613 - 1208780 | ENSBTAG00000045998 | protein_coding |
| ***NDN*** | 21 | 1278161 | 1251601 - 1253837 | ENSBTAG00000002186 | protein_coding |
|  | 21 | 1278161 | 1181795 - 1183106 | ENSBTAG00000059935 | lncRNA |
|  | 21 | 1278161 | 1328347 - 1329354 | ENSBTAG00000071938 | lncRNA |
|  | 21 | 2144071 | 2062791 - 2064779 | ENSBTAG00000049003 | lncRNA |
|  | 21 | 2144071 | 2066249 - 2071852 | ENSBTAG00000069049 | lncRNA |
|  | 21 | 2144071 | 2071916 - 2143580 | ENSBTAG00000070854 | lncRNA |
|  | 21 | 2144071 | 2123329 - 2143495 | ENSBTAG00000066788 | lncRNA |
| ***SNORD1 family*** | 21 | 2144071 | 2094148 - 2094236 | ENSBTAG00000045376 | snoRNA |
|  | 21 | 2144071 | 2220388 - 2220454 | ENSBTAG00000074629 | snoRNA |
| ***ROGDI*** | 25 | 3865404 | 3876344 - 3882573 | ENSBTAG00000016331 | protein_coding |
| ***GLYR1*** | 25 | 3865404 | 3882640 - 3914764 | ENSBTAG00000001731 | protein_coding |
| ***UBN1*** | 25 | 3865404 | 3914961 - 3947858 | ENSBTAG00000001736 | protein_coding |
| ***PPL*** | 25 | 3865404 | 3947863 - 3996586 | ENSBTAG00000002178 | protein_coding |
| ***SEC14L5*** | 25 | 3929914 | 4014672 - 4054833 | ENSBTAG00000007890 | protein_coding |
| ***MGRN1*** | 25 | 3865404 | 3755133 - 3793240 | ENSBTAG00000018999 | protein_coding |
| ***NUDT16L1*** | 25 | 3865404 | 3802680 - 3804334 | ENSBTAG00000006590 | protein_coding |
| ***ANKS3*** | 25 | 3865404 | 3805102 - 3820247 | ENSBTAG00000006593 | protein_coding |
|  | 25 | 3865404 | 3820728 - 3831556 | ENSBTAG00000006595 | protein_coding |
| ***ZNF500*** | 25 | 3865404 | 3832819 - 3844023 | ENSBTAG00000037566 | protein_coding |
| ***SEPTIN12*** | 25 | 3865404 | 3856459 - 3868939 | ENSBTAG00000016330 | protein_coding |
|  | 25 | 3865404 | 3845600 - 3851829 | ENSBTAG00000078992 | lncRNA |
|  | 25 | 3865404 | 3869038 - 3876178 | ENSBTAG00000064535 | lncRNA |
|  | 25 | 3929914 | 4010586 - 4013181 | ENSBTAG00000063531 | lncRNA |
|  | 25 | 3929914 | 4012131 - 4014099 | ENSBTAG00000074271 | lncRNA |
| ***SC365 – Slope*** | |  |  |  |  |
| ***COBLL1*** | 2 | 31663929 | 31429939 - 31597839 | ENSBTAG00000005501 | protein_coding |
| ***GRB14*** | 2 | 31663929 | 31681727 - 31810646 | ENSBTAG00000019291 | protein_coding |
|  | 2 | 31663929 | 31630290 - 31633063 | ENSBTAG00000077981 | lncRNA |
|  | 2 | 31663929 | 31641858 - 31681637 | ENSBTAG00000078676 | lncRNA |
|  | 2 | 31663929 | 31642351 - 31645531 | ENSBTAG00000071435 | lncRNA |
|  | 2 | 31663929 | 31660845 - 31664971 | ENSBTAG00000078032 | lncRNA |
| ***XIRP2*** | 2 | 29725662 | 28988612 - 29679060 | ENSBTAG00000017743 | protein_coding |
|  | 2 | 29725662 | 29713091 - 29714941 | ENSBTAG00000078998 | lncRNA |
|  | 8 | 71848112 | 71934349 - 71940741 | ENSBTAG00000077441 | lncRNA |
| ***RORB*** | 8 | 50615899 | 50700722 - 50763952 | ENSBTAG00000021699 | protein_coding |
|  | 8 | 50615899 | 50667933 - 50668002 | ENSBTAG00000042911 | snoRNA |
|  | 9 | 21153630 | 21142816 - 21150553 | ENSBTAG00000070448 | lncRNA |
|  | 9 | 21153630 | 21154216 - 21155355 | ENSBTAG00000075143 | lncRNA |
|  | 9 | 21153630 | 21220877 - 21232768 | ENSBTAG00000077273 | lncRNA |
|  | 9 | 21153630 | 21232855 - 21257112 | ENSBTAG00000057943 | lncRNA |
| ***SLC9A8*** | 13 | 77958705 | 77899068 - 77973805 | ENSBTAG00000008969 | protein_coding |
| ***SPATA2*** | 13 | 77958705 | 77982558 - 77993066 | ENSBTAG00000018063 | protein_coding |
| ***RNF114*** | 13 | 77958705 | 77993252 - 78017909 | ENSBTAG00000027317 | protein_coding |
| ***SNAI1*** | 13 | 77958705 | 78036441 - 78042568 | ENSBTAG00000014554 | protein_coding |
| ***SNORA70*** | 13 | 77958705 | 78012097 - 78012227 | ENSBTAG00000044591 | snoRNA |
| ***ACER3*** | 15 | 56246427 | 56246786 - 56447013 | ENSBTAG00000017218 | protein_coding |
| ***TSKU*** | 15 | 56246427 | 56178753 - 56204248 | ENSBTAG00000058190 | protein_coding |
|  | 15 | 56246427 | 56174504 - 56176334 | ENSBTAG00000074927 | lncRNA |
|  | 21 | 2144071 | 2062791 - 2064779 | ENSBTAG00000049003 | lncRNA |
|  | 21 | 2144071 | 2066249 - 2071852 | ENSBTAG00000069049 | lncRNA |
|  | 21 | 2144071 | 2071916 - 2143580 | ENSBTAG00000070854 | lncRNA |
|  | 21 | 2144071 | 2123329 - 2143495 | ENSBTAG00000066788 | lncRNA |
| ***SNORD1 family*** | 21 | 2144071 | 2094148 - 2094236 | ENSBTAG00000045376 | snoRNA |
|  | 21 | 2144071 | 2220388 - 2220454 | ENSBTAG00000074629 | snoRNA |
| ***ROGDI*** | 25 | 3865404 | 3876344 - 3882573 | ENSBTAG00000016331 | protein_coding |
| ***GLYR1*** | 25 | 3865404 | 3882640 - 3914764 | ENSBTAG00000001731 | protein_coding |
| ***UBN1*** | 25 | 3865404 | 3914961 - 3947858 | ENSBTAG00000001736 | protein_coding |
| ***PPL*** | 25 | 3865404 | 3947863 - 3996586 | ENSBTAG00000002178 | protein_coding |
| ***SEC14L5*** | 25 | 3929914 | 4014672 - 4054833 | ENSBTAG00000007890 | protein_coding |
| ***MGRN1*** | 25 | 3865404 | 3755133 - 3793240 | ENSBTAG00000018999 | protein_coding |
| ***NUDT16L1*** | 25 | 3865404 | 3802680 - 3804334 | ENSBTAG00000006590 | protein_coding |
| ***ANKS3*** | 25 | 3865404 | 3805102 - 3820247 | ENSBTAG00000006593 | protein_coding |
|  | 25 | 3865404 | 3820728 - 3831556 | ENSBTAG00000006595 | protein_coding |
| ***ZNF500*** | 25 | 3865404 | 3832819 - 3844023 | ENSBTAG00000037566 | protein_coding |
| ***SEPTIN12*** | 25 | 3865404 | 3856459 - 3868939 | ENSBTAG00000016330 | protein_coding |
|  | 25 | 3865404 | 3845600 - 3851829 | ENSBTAG00000078992 | lncRNA |
|  | 25 | 3865404 | 3869038 - 3876178 | ENSBTAG00000064535 | lncRNA |
|  | 25 | 3929914 | 4010586 - 4013181 | ENSBTAG00000063531 | lncRNA |
|  | 25 | 3929914 | 4012131 - 4014099 | ENSBTAG00000074271 | lncRNA |
| ***SC365 - Both*** | |  |  |  |  |
| ***COBLL1*** | 2 | 31663929 | 31429939 - 31597839 | ENSBTAG00000005501 | protein_coding |
| ***GRB14*** | 2 | 31663929 | 31681727 - 31810646 | ENSBTAG00000019291 | protein_coding |
|  | 2 | 31663929 | 31630290 - 31633063 | ENSBTAG00000077981 | lncRNA |
|  | 2 | 31663929 | 31641858 - 31681637 | ENSBTAG00000078676 | lncRNA |
|  | 2 | 31663929 | 31642351 - 31645531 | ENSBTAG00000071435 | lncRNA |
|  | 2 | 31663929 | 31660845 - 31664971 | ENSBTAG00000078032 | lncRNA |
| ***XIRP2*** | 2 | 29725662 | 28988612 - 29679060 | ENSBTAG00000017743 | protein_coding |
|  | 2 | 29725662 | 29713091 - 29714941 | ENSBTAG00000078998 | lncRNA |
|  | 8 | 71848112 | 71934349 - 71940741 | ENSBTAG00000077441 | lncRNA |
| ***RORB*** | 8 | 50615899 | 50700722 - 50763952 | ENSBTAG00000021699 | protein_coding |
|  | 8 | 50615899 | 50667933 - 50668002 | ENSBTAG00000042911 | snoRNA |
|  | 9 | 21153630 | 21142816 - 21150553 | ENSBTAG00000070448 | lncRNA |
|  | 9 | 21153630 | 21154216 - 21155355 | ENSBTAG00000075143 | lncRNA |
|  | 9 | 21153630 | 21220877 - 21232768 | ENSBTAG00000077273 | lncRNA |
|  | 9 | 21153630 | 21232855 - 21257112 | ENSBTAG00000057943 | lncRNA |
| ***SLC9A8*** | 13 | 77958705 | 77899068 - 77973805 | ENSBTAG00000008969 | protein_coding |
| ***SPATA2*** | 13 | 77958705 | 77982558 - 77993066 | ENSBTAG00000018063 | protein_coding |
| ***RNF114*** | 13 | 77958705 | 77993252 - 78017909 | ENSBTAG00000027317 | protein_coding |
| ***SNAI1*** | 13 | 77958705 | 78036441 - 78042568 | ENSBTAG00000014554 | protein_coding |
| ***SNORA70*** | 13 | 77958705 | 78012097 - 78012227 | ENSBTAG00000044591 | snoRNA |
| ***ACER3*** | 15 | 56246427 | 56246786 - 56447013 | ENSBTAG00000017218 | protein_coding |
| ***TSKU*** | 15 | 56246427 | 56178753 - 56204248 | ENSBTAG00000058190 | protein_coding |
|  | 15 | 56246427 | 56174504 - 56176334 | ENSBTAG00000074927 | lncRNA |
|  | 21 | 2144071 | 2062791 - 2064779 | ENSBTAG00000049003 | lncRNA |
|  | 21 | 2144071 | 2066249 - 2071852 | ENSBTAG00000069049 | lncRNA |
|  | 21 | 2144071 | 2071916 - 2143580 | ENSBTAG00000070854 | lncRNA |
|  | 21 | 2144071 | 2123329 - 2143495 | ENSBTAG00000066788 | lncRNA |
| ***SNORD1 family*** | 21 | 2144071 | 2094148 - 2094236 | ENSBTAG00000045376 | snoRNA |
|  | 21 | 2144071 | 2220388 - 2220454 | ENSBTAG00000074629 | snoRNA |
| ***ROGDI*** | 25 | 3865404 | 3876344 - 3882573 | ENSBTAG00000016331 | protein_coding |
| ***GLYR1*** | 25 | 3865404 | 3882640 - 3914764 | ENSBTAG00000001731 | protein_coding |
| ***UBN1*** | 25 | 3865404 | 3914961 - 3947858 | ENSBTAG00000001736 | protein_coding |
| ***PPL*** | 25 | 3865404 | 3947863 - 3996586 | ENSBTAG00000002178 | protein_coding |
| ***SEC14L5*** | 25 | 3929914 | 4014672 - 4054833 | ENSBTAG00000007890 | protein_coding |
| ***MGRN1*** | 25 | 3865404 | 3755133 - 3793240 | ENSBTAG00000018999 | protein_coding |
| ***NUDT16L1*** | 25 | 3865404 | 3802680 - 3804334 | ENSBTAG00000006590 | protein_coding |
| ***ANKS3*** | 25 | 3865404 | 3805102 - 3820247 | ENSBTAG00000006593 | protein_coding |
|  | 25 | 3865404 | 3820728 - 3831556 | ENSBTAG00000006595 | protein_coding |
| ***ZNF500*** | 25 | 3865404 | 3832819 - 3844023 | ENSBTAG00000037566 | protein_coding |
| ***SEPTIN12*** | 25 | 3865404 | 3856459 - 3868939 | ENSBTAG00000016330 | protein_coding |
|  | 25 | 3865404 | 3845600 - 3851829 | ENSBTAG00000078992 | lncRNA |
|  | 25 | 3865404 | 3869038 - 3876178 | ENSBTAG00000064535 | lncRNA |
|  | 25 | 3929914 | 4010586 - 4013181 | ENSBTAG00000063531 | lncRNA |
|  | 25 | 3929914 | 4012131 - 4014099 | ENSBTAG00000074271 | lncRNA |

lncRNA: long non-coding RNA; miRNA: microRNA; snoRNA: small nucleolar RNA; snRNA: small nuclear RNA.

S7. Positional candidate genes located near the regions identified for APM in Nellore cattle.

| **Gene** | **Chromosome** | **Position** | **Start - End Position** | **gene_id** | **gene_biotype** |
| --- | --- | --- | --- | --- | --- |
| **APM - Intercept** | |  |  |  |  |
| ***HSCB*** | 17 | 68042761 | 68012845 - 68024967 | ENSBTAG00000015075 | protein_coding |
| ***TTC28*** | 17 | 68042761 | 67396693 - 67973146 | ENSBTAG00000012193 | protein_coding |
| ***CHEK2*** | 17 | 68042761 | 67975026 - 68012641 | ENSBTAG00000004956 | protein_coding |
| ***XBP1*** | 17 | 68042761 | 68052009 - 68057008 | ENSBTAG00000005970 | protein_coding |
| ***TNIP3*** | 17 | 68042761 | 68140394 - 68209509 | ENSBTAG00000047107 | protein_coding |
|  | 17 | 68042761 | 68077532 - 68079321 | ENSBTAG00000071749 | lncRNA |
|  | 17 | 68042761 | 68093530 - 68096388 | ENSBTAG00000056922 | lncRNA |
| ***CCDC117*** | 17 | 68042761 | 68030505 - 68049483 | ENSBTAG00000003557 | protein_coding |
| **APM – Slope** | |  |  |  |  |
|  | 16 | 69777414 | 69596424 - 69688969 | ENSBTAG00000058856 | lncRNA |
|  | 16 | 69777414 | 69659150 - 69724788 | ENSBTAG00000062426 | lncRNA |
|  | 16 | 69777414 | 69694229 - 69753655 | ENSBTAG00000071485 | lncRNA |
|  | 28 | 38350252 | 38252854 - 38378320 | ENSBTAG00000075687 | lncRNA |

APM: age at puberty in males.

S8. Gene Ontology terms for the genes annotated for SC365 in Nellore cattle.

| **Functional terms** | **Description of function** | **Genes** | |
| --- | --- | --- | --- |
| **SC365 - Intercept** |  | |  |
| **GO:0005730** | Nucleolus | ENSBTAG00000042911,ENSBTAG00000018063,ENSBTAG00000014554,ENSBTAG00000044591,ENSBTAG00000069049,ENSBTAG00000045376,ENSBTAG00000042719,ENSBTAG00000045426,ENSBTAG00000043599,ENSBTAG00000046093,ENSBTAG00000045308,ENSBTAG00000046568,ENSBTAG00000042115,ENSBTAG00000042769,ENSBTAG00000042125 | |
| **GO:0043228** | Membraneless organelle | ENSBTAG00000017743,ENSBTAG00000039530,ENSBTAG00000021949,ENSBTAG00000042911,ENSBTAG00000018063,ENSBTAG00000014554,ENSBTAG00000044591,ENSBTAG00000002186,ENSBTAG00000069049,ENSBTAG00000045376,ENSBTAG00000042719,ENSBTAG00000045426,ENSBTAG00000043599,ENSBTAG00000046093,ENSBTAG00000045308,ENSBTAG00000046568,ENSBTAG00000042115,ENSBTAG00000042769,ENSBTAG00000042125,ENSBTAG00000001731,ENSBTAG00000001736,ENSBTAG00000016330 | |
| **GO:0043232** | Intracellular membraneless organelle | ENSBTAG00000017743,ENSBTAG00000039530,ENSBTAG00000021949,ENSBTAG00000042911,ENSBTAG00000018063,ENSBTAG00000014554,ENSBTAG00000044591,ENSBTAG00000002186,ENSBTAG00000069049,ENSBTAG00000045376,ENSBTAG00000042719,ENSBTAG00000045426,ENSBTAG00000043599,ENSBTAG00000046093,ENSBTAG00000045308,ENSBTAG00000046568,ENSBTAG00000042115,ENSBTAG00000042769,ENSBTAG00000042125,ENSBTAG00000001731,ENSBTAG00000001736,ENSBTAG00000016330 | |
| **GO:0031981** | Nuclear lumen | ENSBTAG00000021699,ENSBTAG00000042911,ENSBTAG00000018063,ENSBTAG00000014554,ENSBTAG00000044591,ENSBTAG00000002186,ENSBTAG00000069049,ENSBTAG00000045376,ENSBTAG00000042719,ENSBTAG00000045426,ENSBTAG00000043599,ENSBTAG00000046093,ENSBTAG00000045308,ENSBTAG00000046568,ENSBTAG00000042115,ENSBTAG00000042769,ENSBTAG00000042125,ENSBTAG00000001736 | |
| **GO:0070013** | Intracellular organelle lumen | ENSBTAG00000021699,ENSBTAG00000042911,ENSBTAG00000018063,ENSBTAG00000014554,ENSBTAG00000044591,ENSBTAG00000002186,ENSBTAG00000069049,ENSBTAG00000045376,ENSBTAG00000042719,ENSBTAG00000045426,ENSBTAG00000043599,ENSBTAG00000046093,ENSBTAG00000045308,ENSBTAG00000046568,ENSBTAG00000042115,ENSBTAG00000042769,ENSBTAG00000042125,ENSBTAG00000001736 | |
| **GO:0043233** | Organelle lumen | ENSBTAG00000021699,ENSBTAG00000042911,ENSBTAG00000018063,ENSBTAG00000014554,ENSBTAG00000044591,ENSBTAG00000002186,ENSBTAG00000069049,ENSBTAG00000045376,ENSBTAG00000042719,ENSBTAG00000045426,ENSBTAG00000043599,ENSBTAG00000046093,ENSBTAG00000045308,ENSBTAG00000046568,ENSBTAG00000042115,ENSBTAG00000042769,ENSBTAG00000042125,ENSBTAG00000001736 | |
| **GO:0031974** | Membrane-enclosed lumen | ENSBTAG00000021699,ENSBTAG00000042911,ENSBTAG00000018063,ENSBTAG00000014554,ENSBTAG00000044591,ENSBTAG00000002186,ENSBTAG00000069049,ENSBTAG00000045376,ENSBTAG00000042719,ENSBTAG00000045426,ENSBTAG00000043599,ENSBTAG00000046093,ENSBTAG00000045308,ENSBTAG00000046568,ENSBTAG00000042115,ENSBTAG00000042769,ENSBTAG00000042125,ENSBTAG00000001736 | |
| **GO:0043226** | Organelle | ENSBTAG00000019291,ENSBTAG00000017743,ENSBTAG00000039530,ENSBTAG00000021949,ENSBTAG00000021699,ENSBTAG00000042911,ENSBTAG00000008969,ENSBTAG00000018063,ENSBTAG00000014554,ENSBTAG00000044591,ENSBTAG00000017218,ENSBTAG00000045998,ENSBTAG00000002186,ENSBTAG00000069049,ENSBTAG00000045376,ENSBTAG00000042719,ENSBTAG00000045426,ENSBTAG00000043599,ENSBTAG00000046093,ENSBTAG00000045308,ENSBTAG00000046568,ENSBTAG00000042115,ENSBTAG00000042769,ENSBTAG00000042125,ENSBTAG00000016331,ENSBTAG00000001731,ENSBTAG00000001736,ENSBTAG00000018999,ENSBTAG00000006593,ENSBTAG00000037566,ENSBTAG00000016330 | |
| **GO:0099160** | Postsynaptic intermediate filament cytoskeleton | ENSBTAG00000021949 | |
| **GO:0005622** | Intracellular anatomical structure | ENSBTAG00000019291,ENSBTAG00000017743,ENSBTAG00000039530,ENSBTAG00000021949,ENSBTAG00000021699,ENSBTAG00000042911,ENSBTAG00000008969,ENSBTAG00000018063,ENSBTAG00000027317,ENSBTAG00000014554,ENSBTAG00000044591,ENSBTAG00000017218,ENSBTAG00000045998,ENSBTAG00000002186,ENSBTAG00000069049,ENSBTAG00000045376,ENSBTAG00000042719,ENSBTAG00000045426,ENSBTAG00000043599,ENSBTAG00000046093,ENSBTAG00000045308,ENSBTAG00000046568,ENSBTAG00000042115,ENSBTAG00000042769,ENSBTAG00000042125,ENSBTAG00000016331,ENSBTAG00000001731,ENSBTAG00000001736,ENSBTAG00000018999,ENSBTAG00000006593,ENSBTAG00000037566,ENSBTAG00000016330 | |
| **GO:0099182** | Presynaptic intermediate filament cytoskeleton | ENSBTAG00000021949 | |
| **GO:0043229** | Intracellular organelle | ENSBTAG00000019291,ENSBTAG00000017743,ENSBTAG00000039530,ENSBTAG00000021949,ENSBTAG00000021699,ENSBTAG00000042911,ENSBTAG00000008969,ENSBTAG00000018063,ENSBTAG00000014554,ENSBTAG00000044591,ENSBTAG00000017218,ENSBTAG00000045998,ENSBTAG00000002186,ENSBTAG00000069049,ENSBTAG00000045376,ENSBTAG00000042719,ENSBTAG00000045426,ENSBTAG00000043599,ENSBTAG00000046093,ENSBTAG00000045308,ENSBTAG00000046568,ENSBTAG00000042115,ENSBTAG00000042769,ENSBTAG00000042125,ENSBTAG00000016331,ENSBTAG00000001731,ENSBTAG00000001736,ENSBTAG00000018999,ENSBTAG00000037566,ENSBTAG00000016330 | |
| **HP:0031169** | Postterm pregnancy | ENSBTAG00000045998,ENSBTAG00000002186 | |
| **HP:0012105** | Occipital cortical atrophy | ENSBTAG00000045998,ENSBTAG00000002186 | |
| **HP:0430147** | Abnormal circulating inhibin B concentration | ENSBTAG00000045998,ENSBTAG00000002186 | |
| **HP:0100739** | Bulimia | ENSBTAG00000045998,ENSBTAG00000002186 | |
| **HP:0009088** | Speech articulation difficulties | ENSBTAG00000005501,ENSBTAG00000045998,ENSBTAG00000002186 | |
| **HP:0031099** | Abnormal circulating inhibin level | ENSBTAG00000045998,ENSBTAG00000002186 | |
| **HP:0031100** | Decreased circulating inhibin B concentration | ENSBTAG00000045998,ENSBTAG00000002186 | |
| **HP:0004039** | Abnormal ulnar metaphysis morphology | ENSBTAG00000045998,ENSBTAG00000002186 | |
| **HP:0012412** | Premature adrenarche | ENSBTAG00000045998,ENSBTAG00000002186 | |
| **HP:0012104** | Parietal cortical atrophy | ENSBTAG00000045998,ENSBTAG00000002186 | |
| **HP:0012166** | Skin-picking | ENSBTAG00000045998,ENSBTAG00000002186 | |
| **HP:0012411** | Premature pubarche | ENSBTAG00000045998,ENSBTAG00000002186 | |
| **HP:0004283** | Narrow palm | ENSBTAG00000045998,ENSBTAG00000002186 | |
| **HP:0011734** | Central adrenal insufficiency | ENSBTAG00000045998,ENSBTAG00000002186 | |
| **HP:0009809** | Abnormal upper limb metaphysis morphology | ENSBTAG00000045998,ENSBTAG00000002186 | |
| **HP:0031507** | Decreased circulating T4 concentration | ENSBTAG00000045998,ENSBTAG00000002186 | |
| **HP:0031505** | Abnormal circulating T4 concentration | ENSBTAG00000045998,ENSBTAG00000002186 | |
| **HP:0000064** | Hypoplastic labia minora | ENSBTAG00000045998,ENSBTAG00000002186 | |
| **HP:0040255** | Aplasia/Hypoplasia of the clitoris | ENSBTAG00000045998,ENSBTAG00000002186 | |
| **HP:0006889** | Intellectual disability, borderline | ENSBTAG00000045998,ENSBTAG00000002186 | |
| **HP:0000060** | Clitoral hypoplasia | ENSBTAG00000045998,ENSBTAG00000002186 | |
| **HP:0031508** | Abnormal circulating thyroid hormone concentration | ENSBTAG00000045998,ENSBTAG00000002186 | |
| **HP:0010829** | Impaired temperature sensation | ENSBTAG00000045998,ENSBTAG00000002186 | |
| **HP:0010627** | Anterior pituitary hypoplasia | ENSBTAG00000045998,ENSBTAG00000002186 | |
| **HP:0030339** | Decreased circulating gonadotropin concentration | ENSBTAG00000045998,ENSBTAG00000002186 | |
| **HP:0012880** | Abnormal labia minora morphology | ENSBTAG00000045998,ENSBTAG00000002186 | |
| **HP:0012650** | Perisylvian polymicrogyria | ENSBTAG00000045998,ENSBTAG00000002186 | |
| **HP:0008770** | Obsessive-compulsive trait | ENSBTAG00000045998,ENSBTAG00000002186 | |
| **HP:0002578** | Gastroparesis | ENSBTAG00000045998,ENSBTAG00000002186 | |
| **HP:0025160** | Abnormal temper tantrums | ENSBTAG00000045998,ENSBTAG00000002186 | |
| **HP:0010625** | Anterior pituitary dysgenesis | ENSBTAG00000045998,ENSBTAG00000002186 | |
| **WP:WP3243** | Amyotrophic lateral sclerosis ALS | ENSBTAG00000039530,ENSBTAG00000021949 | |
| **SC365 - Slope** |  | |  |
| **GO:0005730** | Nucleolus | ENSBTAG00000042911,ENSBTAG00000018063,ENSBTAG00000014554,ENSBTAG00000044591,ENSBTAG00000069049,ENSBTAG00000045376,ENSBTAG00000042719,ENSBTAG00000045426,ENSBTAG00000043599,ENSBTAG00000046093,ENSBTAG00000045308,ENSBTAG00000046568,ENSBTAG00000042115,ENSBTAG00000042769,ENSBTAG00000042125 | |
| **GO:0043228** | Membraneless organelle | ENSBTAG00000017743,ENSBTAG00000042911,ENSBTAG00000018063,ENSBTAG00000014554,ENSBTAG00000044591,ENSBTAG00000069049,ENSBTAG00000045376,ENSBTAG00000042719,ENSBTAG00000045426,ENSBTAG00000043599,ENSBTAG00000046093,ENSBTAG00000045308,ENSBTAG00000046568,ENSBTAG00000042115,ENSBTAG00000042769,ENSBTAG00000042125,ENSBTAG00000001731,ENSBTAG00000001736,ENSBTAG00000016330 | |
| **GO:0043232** | Intracellular membraneless organelle | ENSBTAG00000017743,ENSBTAG00000042911,ENSBTAG00000018063,ENSBTAG00000014554,ENSBTAG00000044591,ENSBTAG00000069049,ENSBTAG00000045376,ENSBTAG00000042719,ENSBTAG00000045426,ENSBTAG00000043599,ENSBTAG00000046093,ENSBTAG00000045308,ENSBTAG00000046568,ENSBTAG00000042115,ENSBTAG00000042769,ENSBTAG00000042125,ENSBTAG00000001731,ENSBTAG00000001736,ENSBTAG00000016330 | |
| **GO:0031981** | Nuclear lumen | ENSBTAG00000021699,ENSBTAG00000042911,ENSBTAG00000018063,ENSBTAG00000014554,ENSBTAG00000044591,ENSBTAG00000069049,ENSBTAG00000045376,ENSBTAG00000042719,ENSBTAG00000045426,ENSBTAG00000043599,ENSBTAG00000046093,ENSBTAG00000045308,ENSBTAG00000046568,ENSBTAG00000042115,ENSBTAG00000042769,ENSBTAG00000042125,ENSBTAG00000001736 | |
| **GO:0031974** | Membrane-enclosed lumen | ENSBTAG00000021699,ENSBTAG00000042911,ENSBTAG00000018063,ENSBTAG00000014554,ENSBTAG00000044591,ENSBTAG00000069049,ENSBTAG00000045376,ENSBTAG00000042719,ENSBTAG00000045426,ENSBTAG00000043599,ENSBTAG00000046093,ENSBTAG00000045308,ENSBTAG00000046568,ENSBTAG00000042115,ENSBTAG00000042769,ENSBTAG00000042125,ENSBTAG00000001736 | |
| **GO:0043233** | Organelle lumen | ENSBTAG00000021699,ENSBTAG00000042911,ENSBTAG00000018063,ENSBTAG00000014554,ENSBTAG00000044591,ENSBTAG00000069049,ENSBTAG00000045376,ENSBTAG00000042719,ENSBTAG00000045426,ENSBTAG00000043599,ENSBTAG00000046093,ENSBTAG00000045308,ENSBTAG00000046568,ENSBTAG00000042115,ENSBTAG00000042769,ENSBTAG00000042125,ENSBTAG00000001736 | |
| **GO:0070013** | Intracellular organelle lumen | ENSBTAG00000021699,ENSBTAG00000042911,ENSBTAG00000018063,ENSBTAG00000014554,ENSBTAG00000044591,ENSBTAG00000069049,ENSBTAG00000045376,ENSBTAG00000042719,ENSBTAG00000045426,ENSBTAG00000043599,ENSBTAG00000046093,ENSBTAG00000045308,ENSBTAG00000046568,ENSBTAG00000042115,ENSBTAG00000042769,ENSBTAG00000042125,ENSBTAG00000001736 | |

Abbreviations: GO: Gene Ontology; BP: Biological Process; CC: Cellular Component; MF: Molecular Function; KEGG: Kyoto Encyclopedia of Genes and Genomes

S9. Gene Ontology terms for the genes annotated for HC30 in Nellore cattle.

| **Functional terms** | **Description of function** | **Genes** |
| --- | --- | --- |
| **HC30 - Intercept** |  |  |
| **GO:0071364** | Cellular response to epidermal growth factor stimulus | ENSBTAG00000012647,ENSBTAG00000013227 |
| **GO:0070849** | Response to epidermal growth factor | ENSBTAG00000012647,ENSBTAG00000013227 |
| **GO:0014033** | Neural crest cell differentiation | ENSBTAG00000012647,ENSBTAG00000013227 |
| **GO:0014032** | Neural crest cell development | ENSBTAG00000012647,ENSBTAG00000013227 |
| **GO:0048864** | Stem cell development | ENSBTAG00000012647,ENSBTAG00000013227 |
| **GO:0035921** | Desmosome disassembly | ENSBTAG00000013227 |
| **GO:0140970** | AIM2 inflammasome complex assembly | ENSBTAG00000050239 |
| **GO:0072046** | Establishment of planar polarity involved in nephron morphogenesis | ENSBTAG00000012647 |
| **GO:0010957** | Negative regulation of vitamin D biosynthetic process | ENSBTAG00000013227 |
| **GO:0070563** | Negative regulation of vitamin D receptor signaling pathway | ENSBTAG00000013227 |
| **GO:0046137** | Negative regulation of vitamin metabolic process | ENSBTAG00000013227 |
| **GO:0150147** | Cell-cell junction disassembly | ENSBTAG00000013227 |
| **GO:0060556** | Regulation of vitamin D biosynthetic process | ENSBTAG00000013227 |
| **GO:0048863** | Stem cell differentiation | ENSBTAG00000012647,ENSBTAG00000013227 |
| **GO:0048762** | Mesenchymal cell differentiation | ENSBTAG00000012647,ENSBTAG00000013227 |
| **GO:0038135** | ERBB2-ERBB4 signaling pathway | ENSBTAG00000012647 |
| **GO:0061026** | Cardiac muscle tissue regeneration | ENSBTAG00000012647 |
| **GO:0021551** | Central nervous system morphogenesis | ENSBTAG00000012647 |
| **GO:0060485** | Mesenchyme development | ENSBTAG00000012647,ENSBTAG00000013227 |
| **GO:0032650** | Regulation of interleukin-1 alpha production | ENSBTAG00000050239 |
| **GO:0030656** | Regulation of vitamin metabolic process | ENSBTAG00000013227 |
| **GO:0043653** | Mitochondrial fragmentation involved in apoptotic process | ENSBTAG00000012647 |
| **GO:0032730** | Positive regulation of interleukin-1 alpha production | ENSBTAG00000050239 |
| **GO:0033629** | Negative regulation of cell adhesion mediated by integrin | ENSBTAG00000013227 |
| **GO:0042368** | Vitamin D biosynthetic process | ENSBTAG00000013227 |
| **GO:0038138** | ERBB4-ERBB4 signaling pathway | ENSBTAG00000012647 |
| **GO:0038130** | ERBB4 signaling pathway | ENSBTAG00000012647 |
| **GO:0032610** | Interleukin-1 alpha production | ENSBTAG00000050239 |
| **GO:0002934** | Desmosome organization | ENSBTAG00000013227 |
| **GO:0021889** | Olfactory bulb interneuron differentiation | ENSBTAG00000012647 |
| **GO:0042362** | Fat-soluble vitamin biosynthetic process | ENSBTAG00000013227 |
| **GO:0070562** | Regulation of vitamin D receptor signaling pathway | ENSBTAG00000013227 |
| **GO:0016540** | Protein autoprocessing | ENSBTAG00000050239 |
| **GO:0060081** | Membrane hyperpolarization | ENSBTAG00000050239 |
| **GO:2000209** | Regulation of anoikis | ENSBTAG00000013227 |
| **GO:0071305** | Cellular response to vitamin D | ENSBTAG00000013227 |
| **GO:2000810** | Regulation of bicellular tight junction assembly | ENSBTAG00000013227 |
| **GO:0070561** | Vitamin D receptor signaling pathway | ENSBTAG00000013227 |
| **GO:2000811** | Negative regulation of anoikis | ENSBTAG00000013227 |
| **GO:0070269** | Pyroptotic inflammatory response | ENSBTAG00000050239 |
| **GO:2000010** | Positive regulation of protein localization to cell surface | ENSBTAG00000012647 |
| **GO:0150146** | Cell junction disassembly | ENSBTAG00000013227 |
| **GO:0061377** | Mammary gland lobule development | ENSBTAG00000012647 |
| **GO:0012501** | Programmed cell death | ENSBTAG00000012647,ENSBTAG00000013227,ENSBTAG00000050239 |
| **GO:0071295** | Cellular response to vitamin | ENSBTAG00000013227 |
| **GO:0060045** | Positive regulation of cardiac muscle cell proliferation | ENSBTAG00000012647 |
| **GO:0060907** | Positive regulation of macrophage cytokine production | ENSBTAG00000050239 |
| **GO:0033198** | Response to ATP | ENSBTAG00000050239 |
| **GO:0033280** | Response to vitamin D | ENSBTAG00000013227 |
| **GO:0034329** | Cell junction assembly | ENSBTAG00000012647,ENSBTAG00000013227 |
| **GO:0038128** | ERBB2 signaling pathway | ENSBTAG00000012647 |
| **GO:0042359** | Vitamin D metabolic process | ENSBTAG00000013227 |
| **GO:0043518** | Negative regulation of DNA damage response, signal transduction by p53 class mediator | ENSBTAG00000013227 |
| **GO:0045939** | Negative regulation of steroid metabolic process | ENSBTAG00000013227 |
| **GO:0010894** | Negative regulation of steroid biosynthetic process | ENSBTAG00000013227 |
| **GO:1902230** | Negative regulation of intrinsic apoptotic signaling pathway in response to DNA damage | ENSBTAG00000013227 |
| **GO:0051882** | Mitochondrial depolarization | ENSBTAG00000050239 |
| **GO:0055023** | Positive regulation of cardiac muscle tissue growth | ENSBTAG00000012647 |
| **GO:0009110** | Vitamin biosynthetic process | ENSBTAG00000013227 |
| **GO:1902531** | Regulation of intracellular signal transduction | ENSBTAG00000012647,ENSBTAG00000013227,ENSBTAG00000050239 |
| **GO:0010839** | Negative regulation of keratinocyte proliferation | ENSBTAG00000013227 |
| **GO:0060644** | Mammary gland epithelial cell differentiation | ENSBTAG00000012647 |
| **GO:0060749** | Mammary gland alveolus development | ENSBTAG00000012647 |
| **GO:0008219** | Cell death | ENSBTAG00000012647,ENSBTAG00000013227,ENSBTAG00000050239 |
| **GO:0008285** | Negative regulation of cell population proliferation | ENSBTAG00000012647,ENSBTAG00000013227 |
| **GO:0006775** | Fat-soluble vitamin metabolic process | ENSBTAG00000013227 |
| **GO:2000008** | Regulation of protein localization to cell surface | ENSBTAG00000012647 |
| **GO:0007595** | Lactation | ENSBTAG00000012647 |
| **GO:0070848** | Response to growth factor | ENSBTAG00000012647,ENSBTAG00000013227 |
| **GO:1901797** | Negative regulation of signal transduction by p53 class mediator | ENSBTAG00000013227 |
| **GO:0033273** | Response to vitamin | ENSBTAG00000013227 |
| **GO:0033993** | Response to lipid | ENSBTAG00000013227,ENSBTAG00000050239 |
| **GO:0043276** | Anoikis | ENSBTAG00000013227 |
| **GO:0099645** | Neurotransmitter receptor localization to postsynaptic specialization membrane | ENSBTAG00000012647 |
| **GO:0099633** | Protein localization to postsynaptic specialization membrane | ENSBTAG00000012647 |
| **GO:0043516** | Regulation of DNA damage response, signal transduction by p53 class mediator | ENSBTAG00000013227 |
| **GO:0046427** | Positive regulation of receptor signaling pathway via JAK-STAT | ENSBTAG00000012647 |
| **GO:0071363** | Cellular response to growth factor stimulus | ENSBTAG00000012647,ENSBTAG00000013227 |
| **GO:0032331** | Negative regulation of chondrocyte differentiation | ENSBTAG00000013227 |
| **GO:0032107** | Regulation of response to nutrient levels | ENSBTAG00000013227 |
| **GO:1902229** | Regulation of intrinsic apoptotic signaling pathway in response to DNA damage | ENSBTAG00000013227 |
| **GO:0060421** | Positive regulation of heart growth | ENSBTAG00000012647 |
| **GO:0061037** | Negative regulation of cartilage development | ENSBTAG00000013227 |
| **GO:0061081** | Positive regulation of myeloid leukocyte cytokine production involved in immune response | ENSBTAG00000050239 |
| **GO:0060043** | Regulation of cardiac muscle cell proliferation | ENSBTAG00000012647 |
| **GO:0021772** | Olfactory bulb development | ENSBTAG00000012647 |
| **GO:0010935** | Regulation of macrophage cytokine production | ENSBTAG00000050239 |
| **GO:1904894** | Positive regulation of receptor signaling pathway via STAT | ENSBTAG00000012647 |
| **GO:0001736** | Establishment of planar polarity | ENSBTAG00000012647 |
| **GO:0010934** | Macrophage cytokine production | ENSBTAG00000050239 |
| **GO:0021988** | Olfactory lobe development | ENSBTAG00000012647 |
| **GO:0007164** | Establishment of tissue polarity | ENSBTAG00000012647 |
| **GO:0031670** | Cellular response to nutrient | ENSBTAG00000013227 |
| **GO:0046622** | Positive regulation of organ growth | ENSBTAG00000012647 |
| **GO:0055021** | Regulation of cardiac muscle tissue growth | ENSBTAG00000012647 |
| **GO:0062237** | Protein localization to postsynapse | ENSBTAG00000012647 |
| **GO:0034330** | Cell junction organization | ENSBTAG00000012647,ENSBTAG00000013227 |
| **GO:0001817** | Regulation of cytokine production | ENSBTAG00000013227,ENSBTAG00000050239 |
| **GO:0001816** | Cytokine production | ENSBTAG00000013227,ENSBTAG00000050239 |
| **GO:0001755** | Neural crest cell migration | ENSBTAG00000012647 |
| **GO:1903539** | Protein localization to postsynaptic membrane | ENSBTAG00000012647 |
| **GO:0010837** | Regulation of keratinocyte proliferation | ENSBTAG00000013227 |
| **GO:0060420** | Regulation of heart growth | ENSBTAG00000012647 |
| **GO:0090497** | Mesenchymal cell migration | ENSBTAG00000012647 |
| **GO:0060038** | Cardiac muscle cell proliferation | ENSBTAG00000012647 |
| **GO:0061082** | Myeloid leukocyte cytokine production | ENSBTAG00000050239 |
| **GO:0072557** | IPAF inflammasome complex | ENSBTAG00000050239 |
| **GO:0061702** | Canonical inflammasome complex | ENSBTAG00000050239 |
| **GO:0004197** | Cysteine-type endopeptidase activity | ENSBTAG00000050239,ENSBTAG00000020884 |
| **GO:0038131** | Neuregulin receptor activity | ENSBTAG00000012647 |
| **GO:0008234** | Cysteine-type peptidase activity | ENSBTAG00000050239,ENSBTAG00000020884 |
| **GO:0005006** | Epidermal growth factor receptor activity | ENSBTAG00000012647 |
| **GO:0050811** | GABA receptor binding | ENSBTAG00000012647 |
| **GO:0004175** | Endopeptidase activity | ENSBTAG00000050239,ENSBTAG00000020884 |
| **GO:0005154** | Epidermal growth factor receptor binding | ENSBTAG00000012647 |
| **KEGG:04621** | NOD-like receptor signaling pathway | ENSBTAG00000050239,ENSBTAG00000020884 |
| **KEGG:04613** | Neutrophil extracellular trap formation | ENSBTAG00000050239,ENSBTAG00000020884 |
| **KEGG:05132** | Salmonella infection | ENSBTAG00000050239,ENSBTAG00000020884 |
| **REAC:R-BTA-1253288** | Downregulation of ERBB4 signaling | ENSBTAG00000012647 |
| **REAC:R-BTA-8847993** | ERBB2 Activates PTK6 Signaling | ENSBTAG00000012647 |
| **REAC:R-BTA-6785631** | ERBB2 Regulates Cell Motility | ENSBTAG00000012647 |
| **REAC:R-BTA-1963640** | GRB2 events in ERBB2 signaling | ENSBTAG00000012647 |
| **REAC:R-BTA-448706** | Interleukin-1 processing | ENSBTAG00000050239 |
| **REAC:R-BTA-1251985** | Nuclear signaling by ERBB4 | ENSBTAG00000012647 |
| **REAC:R-BTA-1963642** | PI3K events in ERBB2 signaling | ENSBTAG00000012647 |
| **REAC:R-BTA-1250342** | PI3K events in ERBB4 signaling | ENSBTAG00000012647 |
| **REAC:R-BTA-1250196** | SHC1 events in ERBB2 signaling | ENSBTAG00000012647 |
| **REAC:R-BTA-9008059** | Interleukin-37 signaling | ENSBTAG00000050239 |
| **REAC:R-BTA-1250347** | SHC1 events in ERBB4 signaling | ENSBTAG00000012647 |
| **REAC:R-BTA-5620971** | Pyroptosis | ENSBTAG00000050239 |
| **REAC:R-BTA-8863795** | Downregulation of ERBB2 signaling | ENSBTAG00000012647 |
| **REAC:R-BTA-1236394** | Signaling by ERBB4 | ENSBTAG00000012647 |
| **REAC:R-BTA-1227986** | Signaling by ERBB2 | ENSBTAG00000012647 |
| **REAC:R-BTA-8848021** | Signaling by PTK6 | ENSBTAG00000012647 |
| **REAC:R-BTA-9006927** | Signaling by Non-Receptor Tyrosine Kinases | ENSBTAG00000012647 |
| **REAC:R-BTA-5218859** | Regulated Necrosis | ENSBTAG00000050239 |
| **REAC:R-BTA-9018519** | Estrogen-dependent gene expression | ENSBTAG00000012647 |
| **REAC:R-BTA-199418** | Negative regulation of the PI3K/AKT network | ENSBTAG00000012647 |
| **REAC:R-BTA-6811558** | PI5P, PP2A and IER3 Regulate PI3K/AKT Signaling | ENSBTAG00000012647 |
| **REAC:R-BTA-446652** | Interleukin-1 family signaling | ENSBTAG00000050239 |
| **REAC:R-BTA-5357801** | Programmed Cell Death | ENSBTAG00000050239 |
| **WP:WP3147** | NOD pathway | ENSBTAG00000020884 |
| **WP:WP3148** | Apoptosis modulation and signaling | ENSBTAG00000020884 |
| **WP:WP1018** | Apoptosis | ENSBTAG00000020884 |
| **HC30 - Slope** |  |  |
| **GO:0030853** | Negative regulation of granulocyte differentiation | ENSBTAG00000023179 |
| **GO:0030854** | Positive regulation of granulocyte differentiation | ENSBTAG00000023179 |
| **GO:0031665** | Negative regulation of lipopolysaccharide-mediated signaling pathway | ENSBTAG00000023179 |
| **GO:0034086** | Maintenance of sister chromatid cohesion | ENSBTAG00000009394 |
| **GO:0034088** | Maintenance of mitotic sister chromatid cohesion | ENSBTAG00000009394 |
| **GO:0034091** | Regulation of maintenance of sister chromatid cohesion | ENSBTAG00000009394 |
| **GO:0034093** | Positive regulation of maintenance of sister chromatid cohesion | ENSBTAG00000009394 |
| **GO:0034182** | Regulation of maintenance of mitotic sister chromatid cohesion | ENSBTAG00000009394 |
| **GO:0034184** | Positive regulation of maintenance of mitotic sister chromatid cohesion | ENSBTAG00000009394 |
| **GO:0030223** | Neutrophil differentiation | ENSBTAG00000023179 |
| **GO:0030222** | Eosinophil differentiation | ENSBTAG00000023179 |
| **GO:0045645** | Positive regulation of eosinophil differentiation | ENSBTAG00000023179 |
| **GO:0000722** | Telomere maintenance via recombination | ENSBTAG00000009394 |
| **GO:0045651** | Positive regulation of macrophage differentiation | ENSBTAG00000023179 |
| **GO:0045658** | Regulation of neutrophil differentiation | ENSBTAG00000023179 |
| **GO:0045659** | Negative regulation of neutrophil differentiation | ENSBTAG00000023179 |
| **GO:0045842** | Positive regulation of mitotic metaphase/anaphase transition | ENSBTAG00000009394 |
| **GO:0045876** | Positive regulation of sister chromatid cohesion | ENSBTAG00000009394 |
| **GO:1901970** | Positive regulation of mitotic sister chromatid separation | ENSBTAG00000009394 |
| **GO:1902101** | Positive regulation of metaphase/anaphase transition of cell cycle | ENSBTAG00000009394 |
| **GO:0045643** | Regulation of eosinophil differentiation | ENSBTAG00000023179 |
| **GO:0007063** | Regulation of sister chromatid cohesion | ENSBTAG00000009394 |
| **GO:0031664** | Regulation of lipopolysaccharide-mediated signaling pathway | ENSBTAG00000023179 |
| **GO:0030852** | Regulation of granulocyte differentiation | ENSBTAG00000023179 |
| **GO:0006312** | Mitotic recombination | ENSBTAG00000009394 |
| **GO:0014912** | Negative regulation of smooth muscle cell migration | ENSBTAG00000023179 |
| **GO:0045649** | Regulation of macrophage differentiation | ENSBTAG00000023179 |
| **GO:1905820** | Positive regulation of chromosome separation | ENSBTAG00000009394 |
| **GO:0007064** | Mitotic sister chromatid cohesion | ENSBTAG00000009394 |
| **GO:0045840** | Positive regulation of mitotic nuclear division | ENSBTAG00000009394 |
| **GO:0014910** | Regulation of smooth muscle cell migration | ENSBTAG00000023179 |
| **GO:0019538** | Protein metabolic process | ENSBTAG00000023179,ENSBTAG00000009394,ENSBTAG00000065404 |
| **GO:0031663** | Lipopolysaccharide-mediated signaling pathway | ENSBTAG00000023179 |
| **GO:0048662** | Negative regulation of smooth muscle cell proliferation | ENSBTAG00000023179 |
| **GO:0002762** | Negative regulation of myeloid leukocyte differentiation | ENSBTAG00000023179 |
| **GO:0014909** | Smooth muscle cell migration | ENSBTAG00000023179 |
| **GO:0030851** | Granulocyte differentiation | ENSBTAG00000023179 |
| **GO:0016925** | Protein sumoylation | ENSBTAG00000009394 |
| **GO:0051785** | Positive regulation of nuclear division | ENSBTAG00000009394 |
| **GO:0010965** | Regulation of mitotic sister chromatid separation | ENSBTAG00000009394 |
| **GO:0007062** | Sister chromatid cohesion | ENSBTAG00000009394 |
| **GO:0051306** | Mitotic sister chromatid separation | ENSBTAG00000009394 |
| **GO:0030225** | Macrophage differentiation | ENSBTAG00000023179 |
| **GO:1902099** | Regulation of metaphase/anaphase transition of cell cycle | ENSBTAG00000009394 |
| **GO:0007091** | Metaphase/anaphase transition of mitotic cell cycle | ENSBTAG00000009394 |
| **GO:0030071** | Regulation of mitotic metaphase/anaphase transition | ENSBTAG00000009394 |
| **GO:0014812** | Muscle cell migration | ENSBTAG00000023179 |
| **GO:0002763** | Positive regulation of myeloid leukocyte differentiation | ENSBTAG00000023179 |
| **GO:0044784** | Metaphase/anaphase transition of cell cycle | ENSBTAG00000009394 |
| **GO:0018205** | Peptidyl-lysine modification | ENSBTAG00000009394 |
| **GO:1905818** | Regulation of chromosome separation | ENSBTAG00000009394 |
| **GO:0032436** | Positive regulation of proteasomal ubiquitin-dependent protein catabolic process | ENSBTAG00000023179 |
| **GO:0030915** | Smc5-Smc6 complex | ENSBTAG00000009394 |
| **GO:0106068** | SUMO ligase complex | ENSBTAG00000009394 |
| **GO:0004842** | Ubiquitin-protein transferase activity | ENSBTAG00000023179,ENSBTAG00000009394 |
| **GO:0016755** | Aminoacyltransferase activity | ENSBTAG00000023179,ENSBTAG00000009394 |
| **GO:0019787** | Ubiquitin-like protein transferase activity | ENSBTAG00000023179,ENSBTAG00000009394 |
| **GO:0031434** | Mitogen-activated protein kinase kinase binding | ENSBTAG00000023179 |
| **GO:0061665** | SUMO ligase activity | ENSBTAG00000009394 |
| **GO:0019789** | SUMO transferase activity | ENSBTAG00000009394 |
| **GO:0016746** | Acyltransferase activity | ENSBTAG00000023179,ENSBTAG00000009394 |
| **GO:0055106** | Ubiquitin-protein transferase regulator activity | ENSBTAG00000023179 |
| **REAC:R-BTA-3108214** | SUMOylation of DNA damage response and repair proteins | ENSBTAG00000009394 |
| **REAC:R-BTA-3108232** | SUMO E3 ligases SUMOylate target proteins | ENSBTAG00000009394 |
| **REAC:R-BTA-2990846** | SUMOylation | ENSBTAG00000009394 |

Abbreviations: GO: Gene Ontology; BP: Biological Process; CC: Cellular Component; MF: Molecular Function; KEGG: Kyoto Encyclopedia of Genes and Genomes
